# New Apterodontinae (Hyaenodontida) from the Eocene Locality of Dur At-Talah (Libya): Systematic, Paleoecological and Phylogenetical Implications

**DOI:** 10.1371/journal.pone.0049054

**Published:** 2012-11-21

**Authors:** Camille Grohé, Michael Morlo, Yaowalak Chaimanee, Cécile Blondel, Pauline Coster, Xavier Valentin, Mustapha Salem, Awad A. Bilal, Jean-Jacques Jaeger, Michel Brunet

**Affiliations:** 1 Institut de Paléoprimatologie, Paléontologie Humaine: Évolution et Paléoenvironnents, UMR CNRS 7262, Université de Poitiers, Poitiers, France; 2 Abt. Messelforschung, Forschunginstitut Senckenberg, Frankfurt, Germany; 3 Section of Vertebrate Paleontology, Carnegie Museum of Natural History, Pittsburgh, Pennsylvania, United States of America; 4 Geology Department, University of Al-Fateh, Tripoli, Libya; 5 Geology Department, University of Garyoumis, Bengahzi, Libya; Ecole Normale Supérieure de Lyon, France

## Abstract

The African Hyaenodontida, mainly known from the Late Eocene and Early Oligocene Fayum depression in Egypt, show a very poor diversity in oldest Paleogene localities. Here we report new hyaenodontidans found in the late Middle Eocene deposits of Dur At-Talah (Central Libya), known to have recorded the earliest radiation of African anthropoids. The new hyaenodontidan remains are represented by dental and postcranial specimens comprising the historical material discovered by R.J.G. Savage in the last century and that of the recent Franco-Libyan campaigns. This material includes two apterodontines, in particular a subcomplete skeleton of *Apterodon langebadreae* nov. sp., bringing new postcranial elements to the fossil record of the genus *Apterodon*. Anatomical analysis of the postcranial remains of Dur At-Talah suggests a semi-aquatic lifestyle for *Apterodon*, a completely unusual locomotion pattern among hyaenodontidans. We also perform the first cladistic analysis of hyaenodontidans including apterodontines: *Apterodon* and *Quasiapterodon* appear close relatives to “hyainailourines”, in particular to the African Oligo-Miocene *Metasinopa* species. *Apterodon langebadreae* nov. sp. could be the most primitive species of the genus, confirming an African origin of the Apterodontinae and a further dispersion event to Europe before the early Oligocene. These data enhance our knowledge of early hyaenodontidan diversification into Africa and underline how crucial is the understanding of their evolutionary history for the improvement of Paleogene paleobiogeographic scenarii.

## Introduction

**Figure 1:**
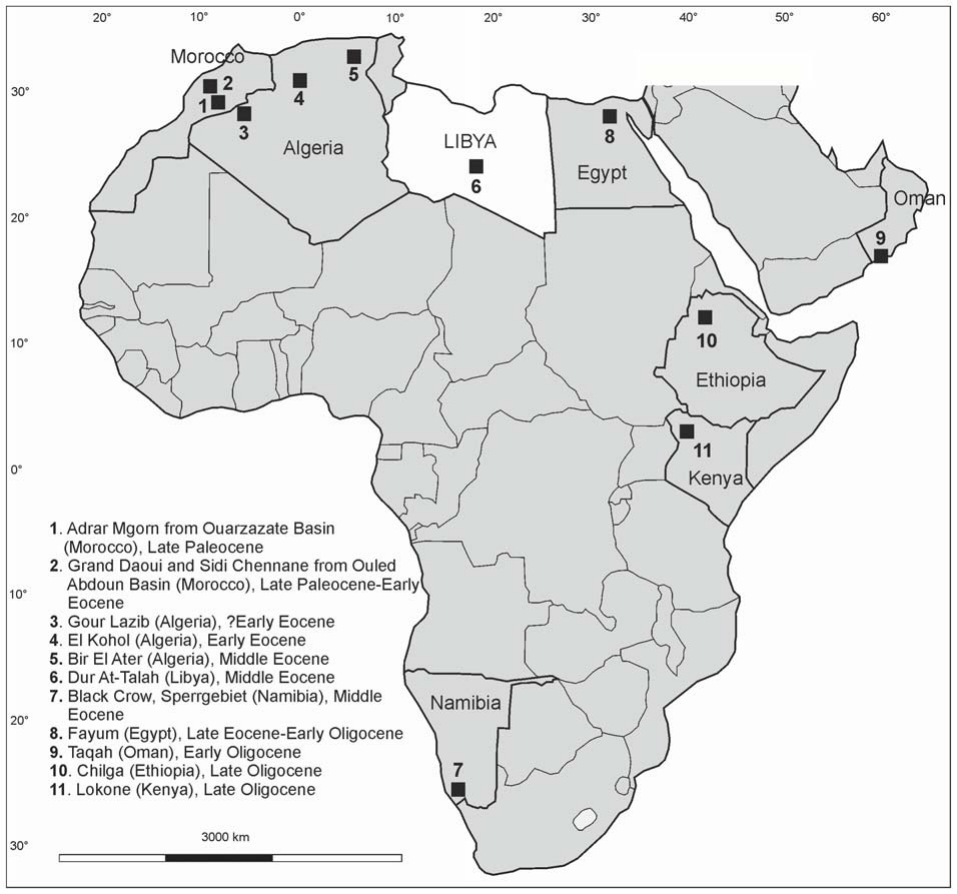
Map situated the Paleogene afro-arabian localities bearing hyaenodontidan fossils.

The Hyaenodontida constitute the most abundant carnivorous placental mammals of Africa during the Paleogene. They are distributed into North America, Eurasia and Africa during the Paleocene-Miocene time. First African hyaenodontidan occurrences are recorded in the Late Paleocene of the Ouled Abdoun and Ouarzazate Basins of Morocco [1]–[5]. More recent Paleogene species from the Afro-Arabian continent are recorded in the North African and Arabian Eocene localities: Taqah in the Sultanate of Oman [6], El Kohol, the Gour Lazib and Bir El Ater in Algeria [7]–[11] and the Fayum in Egypt, where this diversity is the most important [12]–[18]. Two species from the Eocene of Namibia are also reported to hyaenodontidans [19]. The Oligocene fossil record, outside that of the Fayum, is restricted to the East African sites of Chilga (Ethiopia) and Lokone (Kenya) [20], [21] (see Figure 1 for the Afro-Arabian geographical distributions of Paleogene hyaenodontidans).

The Hyaenodontida (Van Valen, 1967) [22], previously known as a family within “Creodonta”, include seven “subfamilies” (i.e., Koholiinae, Limnocyoninae, “Proviverrinae”, Hyainailourinae, Teratodontinae, Apterodontinae and Hyaenodontinae) [7], [23]–[26]. The Apterodontinae represent a peculiar subfamily [13], [14], [18], [27]–[30] which phylogenetical relationships are still unclear. This subfamily includes only two genera, *Apterodon* and *Quasiapterodon*, recovered from the Late Eocene and Oligocene of Africa (Egypt, Kenya) and of Europe (France, Germany) [18], [20], [25], [29]–[32].

The Eocene Dur At-Talah deposits, in Central Libya, yielded the earliest radiation of African anthropoids [33] and provided one of the richest Paleogene faunas of North Africa. This fauna includes several aquatic and terrestrial vertebrates (fish, turtles, crocodiles, squamates, birds) and several marsupial and placental mammals (proboscideans, sirenians, macroscelids, hyracoids, rodents, chiropteres, primates, arsinoitheres, cetaceans, and hyaenodontidans) [33]–[40]. The first hyaenodontidan remains have been found during the fieldwork conducted by R.J.G. Savage in 1968 and 1969 and simply referred to “Creodonta” indet. [34]–[36]. Recent paleontological prospections by a Franco-Libyan team (iPHEP, University of Poitiers, France, and University of Al-Fateh, Tripoli, Libya) have provided new hyaenodontidan fossils at Dur At-Talah, belonging to the Apterodontinae subfamily. In this study, we describe the historical and the new dental and postcranial material of hyaenodontidans from this outcrop, we provide ecological clues for their locomotory styles, and we attempt to propose phylogenetical relationships of Apterodontinae within Hyaenodontida.

### Geological setting

The Dur At-Talah is located in Central Libya, to the northern limit of the Sarir Tibisti Basin and to the southeastern corner of Al Haruj basalt massif. The Dur At-Talah sequence is exposed throughout a 150 km long escarpment, running East-West from roughly Lat. 25°45′ at Long. 17°50′ to 19°15′, with a maximum height of 100 m. It is originally divided into three lithological units from base to top: the Evaporite, the Idam and the Sarir Units [36] (see [33], [41], [42] for alternative stratigraphical units). These deposits show a transition from marine to fluvial environments as an evidence of a Paleogene northwards regressive episode [36], [42].

The successive French and English paleontological expeditions at Dur At-Talah during the 1950s and 1960s [34]–[36], [43]–[45] and the recent American and Franco-libyan fieldworks [33], [40], [46] have permitted the discovery of numerous fossil vertebrates. The main terrestrial vertebrate-bearing localities of the sequence have been reported from the Idam Unit, which corresponds to a marine dominated environment with freshwater ingression [36], [42]. West of the escarpment, R.J.G. Savage collected in 1968 and 1969 about thirty specimens of hyaenodontidans, from lenses of conglomerate of the Idam Unit. These remains were associated with a rich vertebrate fauna of fish, turtles, crocodiles and small mammals, such as rodents and hyracoids [34], [35]. In 2007 and 2009, the Franco-Libyan expeditions (University of Poitiers and University of Al-Fateh, in Tripoli) led to the discovery of additional carnivorous remains from the west to the middle part of the cliff. This material comes from the Idam Unit too.

The age of the Dur At-Talah sequence has been firstly estimated to Upper Eocene and Lower Oligocene by analogy with the Fayum Formations (Egypt) and by stratigraphic relationship with other Paleogene sections of Libya [36]. The generic composition similarities of the fauna seemed to support the contemporaneity of the Fayum and the Dur At-Talah assemblages (e.g., [36], [42], [46]–[49]). Recent biostratigraphical studies, based notably on the rodent and primate faunas, combined with magnetostratigraphic data rather suggest a Late Bartonian age for the Dur At-Talah fossiliferous strata [33], [40].

## Materials and Methods

The material described is temporarily stored at the Institut de Paléoprimatologie et Paléontologie humaine: Évolution et paléoenvironnement **(iPHEP)**, France, and will be returned to the Natural History Museum of London, United Kingdom (**NHM; BMNH**, British Museum of Natural History for fossil references) and to the University of Al-Fateh, Tripoli, Libya (**DT** for fossil references). The recent Franco-Libyan fieldworks have been realized in cooperation with the University of Poitiers, France, and the University of Al-Fateh in Tripoli, Libya. The comparative material of apterodontines comes from the following institutions: American Museum of Natural History, New York, New York **(AMNH)**, Bayerische Staatssammlung für Paläontologie und historische Geologie, Munich, Germany **(BSPG)**, Cairo Geological Museum, Cairo, Egypt **(CGM)**, Hessisches Landesmuseum Darmstadt, Germany **(HLMD)**, Muséum d'Histoire Naturelle de Paris, France **(MNHN)**, Staatliches Museum für Naturkunde Stuttgart, Germany **(SMNS)**, University of California Museum of Paleontology, Berkeley, California **(UCMP)**, Peabody Museum of Natural History, Yale University, New Haven, Connecticut **(YPM)**.

Dental measurements, when from first hand (precised in the table captions), were taken with a digital caliper to the nearest 0.1 mm following Gingerich and Deutsch [50]. Postcranial measurements were taken with ImageJ based on specimen photos. The dental terminology follows Szalay [51].

### Nomenclatural Acts

The electronic edition of this article conforms to the requirements of the amended International Code of Zoological Nomenclature, and hence the new names contained herein are available under that Code from the electronic edition of this article. This published work and the nomenclatural acts it contains have been registered in ZooBank, the online registration system for the ICZN. The ZooBank LSIDs (Life Science Identifiers) can be resolved and the associated information viewed through any standard web browser by appending the LSID to the prefix “http://zoobank.org/”. The LSID for this publication is: urn:lsid:zoobank.org:pub:2F705A08-0E55-41E2-B869-E96C37FCB307. The electronic edition of this work was published in a journal with an ISSN, and has been archived and is available from the following digital repositories: PubMed Central, LOCKSS.

## Results and Discussion

### Systematic descriptions

Order Hyaenodontida (Van Valen, 1967) [22]

The diphyly of the former order “Creodonta” has been proposed by Van Valen [22] and generally accepted and followed in later publications (e.g., [23], [52]–[54]). We use the term Hyaenodontida as employed by Solé [55], [56] who performed cladistic analyses of the group (see [57] for the Oxyaenidan fossil record).

Family Hyaenodontidae (Leidy, 1869) [58]

Apterodontinae (Szalay, 1967) [27]

#### Type genus

*Apterodon* Fischer, 1880 [59]

#### Other included genus

*Quasiapterodon* Lavrov, 1999 [60] (see [25] for a discussion of this genus)

#### Diagnosis (Osborn, 1909 [13]: pp. 417)

Lower molars with paraconid, protoconid, and more or less complex talonid. Upper molars triangular, as in *Tritemnodon* and *Sinopa*, with protocone prominent, subequal paracone, metacone, and styles. Teeth tubercular rather than sectorial.

***Apterodon*** Fischer, 1880 [59]

#### Type species

*Apterodon gaudryi* Fischer, 1880 [59] ( = *A. flonheimensis* Andreae, 1887 [32] in [31])

#### Other included species

*A. macrognathus* (Andrews, 1904) [13], *A. altidens* Schlosser, 1910 [15], *A. saghensis* Simons & Gingerich, 1976 [61], *A. intermedius* Lange-Badré & Böhme, 2005 [30].

#### Emended diagnosis

m3 paraconid more lingual than m2 one, molar metaconids vestigial or absent, labial cingulid arising behind hypoconid in m2–m3, sometimes forming a hypoconulid, lower molars with a crestiform entoconid, m3 talonid basin absent, P4 with protocone lobe, tritubercular upper molars, M1 paracone and metacone of similar height. *Apterodon* differs from *Quasiapterodon* by its larger size, shorter p4 talonid, shorter m1 relative to m2–m3, m3 paraconid more lingual compared to m2 rather than similarly positioned.

#### Remarks

The genus *Apterodon* had been removed from “Creodonta” and was assigned to mesonychid condylarths by Van Valen [62]. Szalay [27] reassigned it to Hyaenodontidae and created a new tribe for this genus: the “Apterodontini”, within the subfamily Hyaenodontinae *s.l.*. Holroyd [18] elevated it to a subfamilial rank. *Apterodon rauenbergensis* from the Early Oligocene of Rauenberg (Germany) was described by Frey *et al.* [63] based on a worn mandibule, on which basal crowns indicated premolar and molar proportions different from those of the other *Apterodon* species. Moreover, several features such as a very short and high mandible, a similar length of p4, m1 and m2, a molariform p4 and a mandibular symphysis extending under the p2, renders unlikely the assignment of this specimen to the genus *Apterodon*.

**Figure 2:**
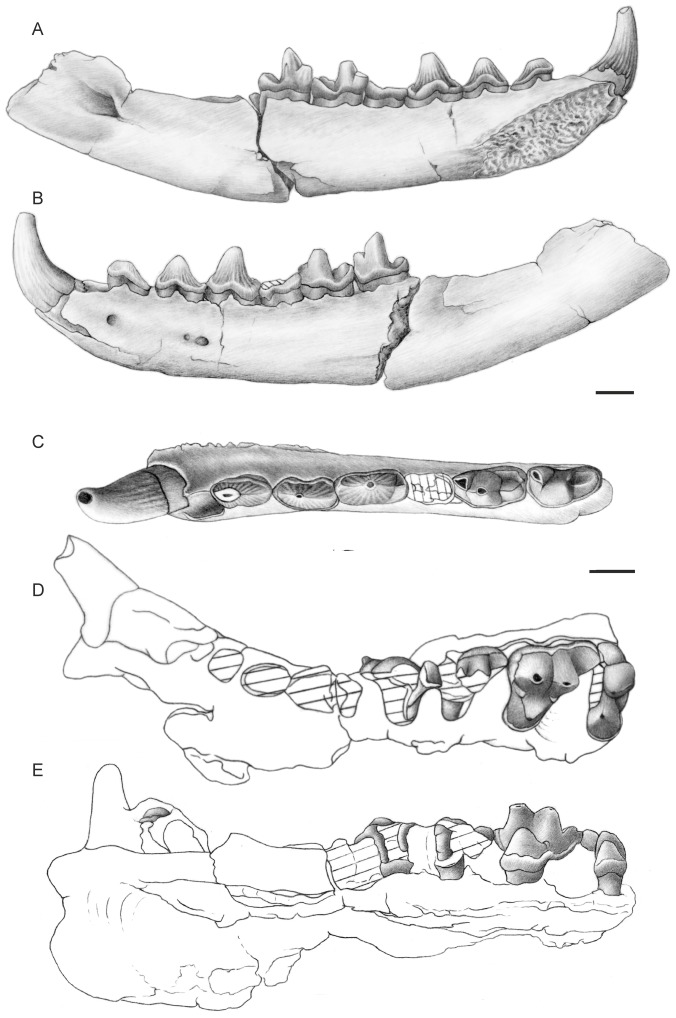
Dental material of *Apterodon langebadreae* nov. sp. BMNH M 85298, left mandible with c, p2-m3 and alveoli of i1–i3 and p1, in lingual (A), in labial (B), and in occlusal (C) views; BMNH M 85300, left maxilla with C-P1, P4-M3 and alveoli of P2–P3, in occlusal (D) and lingual (E) views. Scale = 1 cm. Drawings by Sabine Riffaut.

**Figure 3:**
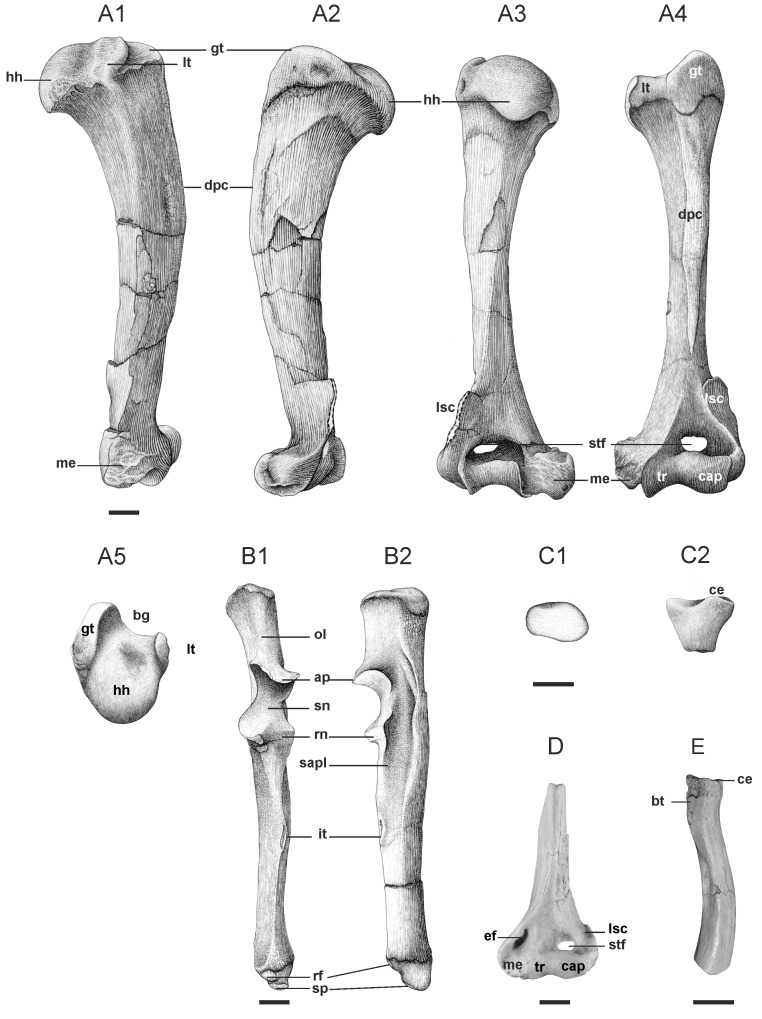
Forelimb postcranium of Dur At-Talah hyaenodontidans. **A, B, C,** ***Apterodon langebadreae*** **nov. sp.** and **D,** **E,** ***Apterodon*** **indet.**; **A,** BMNH M 85318 Left humerus in medial (A1), lateral (A2), ventral (A3), dorsal (A4) and proximal (A5) views; **B,** BMNH M 85322 Left ulna in dorsal (B1) and lateral (B2) views; **C,** BMNH M 85323 Proximal right radius in proximal (C1) and dorsal (C2) views; **D,** DT24-13 Distal left humerus in dorsal view; **E,** DT24-14 Proximal right radius in lateral view. **Abbreviations: ap,** anconeal process; **bg,** bicipital groove; **bt,** bicipital tuberosity; **cap,** capitulum; **ce,** capitular eminence; **dpc,** deltopectoral crest; **ef,** entepicondylar foramen; **gt,** greater tuberosity; **hh,** humeral head; **it,** interosseous tubercle; **lsc,** lateral supinator crest; **lt,** lesser tuberosity; **me,** medial epicondyle; **ol,** olecranon; **rf,** radial facet; **rn,** radial notch; **sapl,** scar for the *M. abductor pollicis longus*; **sn,** semilunar notch; **sp,** styloid process; **stf,** supratrochlear foramen; **tr,** trochlea. Scale = 1 cm. Drawings by Sabine Riffaut.

**Figure 4:**
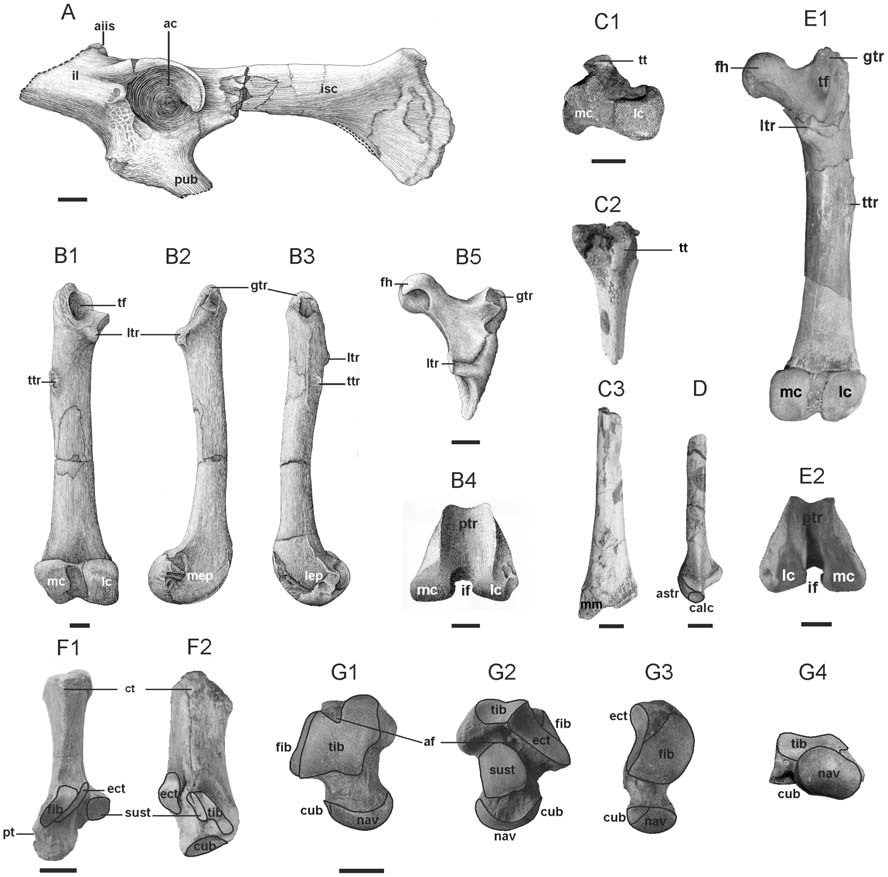
Hindlimb postcranium of Dur At-Talah hyaenodontidans. **A, B, C, D,** ***Apterodon langebadreae*** **nov. sp.** and **E, F, G,** ***Apterodon*** **indet.**; **A,** BMNH M 85332 Left innominate in lateral view; **B,** BMNH M 85315 Left femur in dorsal (B1), medial (B2), lateral (B3) and distal (B4) views and BMNH M 85317 Right proximal femur in dorsal view (B5); **C,** BMNH M 85319 Proximal right tibia in proximal (C1) and dorsal (C2) views and BMNH M 85320 Distal right tibia in ventral view (C3); **D,** BMNH M 85331 Distal right fibula in ventral view; **E,** DT24-6 Right femur in ventral (E1) and distal (E2) views; **F,** DT24-4 Right calcaneus in dorsal (F1) and medial (F2) views; **G,** DT24-5 Right astragalus in dorsal (G1), ventral (G2), lateral (G3) and proximal (H4) views. **Abbreviations: ac,** acetabulum; **af,** astragalar foramen; **aiis,** anterior inferior iliac spine; **astr,** astragalar facet; **calc,** calcaneal facet; **ct,** calcaneal tuber; **cub,** cuboid facet; **ect,** ectal facet; **fh,** femoral head; **fib,** fibular facet; **gtr,** great trochanter; **if,** intercondylar fossa; **il,** ilium; **isc,** ischium; **lc,** lateral condyle; **lep,** lateral epicondyle; **ltr,** lesser trochanter; **mc,** medial condyle; **mep,** medial epicondyle; **mm,** medial malleolus; **nav,** navicular facet; **pt,** peroneal tubercle; **ptr,** patellar trochlea; **pub,** pubis; **sust,** sustentacular facet; **tf,** trochanteric fossa; **tib,** tibial facet; **tt,** tibial tuberosity; **ttr,** third trochanter. Scale = 1 cm. Drawings by Sabine Riffaut.

*Apterodon langebadreae* nov. sp.

urn:lsid:zoobank.org:act:A35DB3C9-4F3A-44CB-9245-F31CB34F62BD

(Figures 2, 3, 4, Tables 1–2)

**Table 1:** Measurements of lower dentition of Apterodontinae (in mm).

	Dbm3	Wbm3	m3	m2	m1	p4	p3	p2	c
***Apterodon langebadreae***									
BMNH M 85298 (holotype)^3^	25.3	11.8	14.2×8,3	13.8×8.2	10.5×6.5	14.5×7.1	13×6.4	11.5×5.4	11×9.4
DT25-1^3^	20.8	7.9		14.3×8	10.8×5	13.3×6.4			
***Apterodon*** ** indet.**									
DT24-1^3^	20.5	10.7	12×7.1						
DT24-2^3^	19.5	10.6				10.9×?			
***A. macrognathus***									
BMNH M 8880 (holotype cast)^3^	33.3	15	17.4×?	16.1×9.9	12.8×7.02	16.1×9.7	14.7×8.5	12.9×7.1	
BMNH M 8873^3^	33.3	12.9	18.4×10.1	17×9.8	12.9×6.4				
BMNH M 8437a^3^	32.9	13.8							
BMNH M 8437b^3^	32.5	14.2				13.2×7.1	11.3×5.9		
BMNH M 8436^3^	32.3	12.5							
BMNH M 10348^3^	31	12.4	15.6×8.4	14.3*×7.6					
BSPG 1905 XIII 9^3^	39	15.5	18.5×10.4	18.1×9.9	13.1×7.3	17.9×9.2		13.1×7.7	19×16.4
SMNS 12643^3^	33.2	13.5	13.9×8.3	14.4×8.7		15.3×8.9	10.7×6.4	12.1×7.2	16.5×12
SMNS 11950^3^	34.5	13.2	15.8×8.6	15.7*×9		15.1×8			
UCMP 62218^1^					13.4*×7*	18.7×8.9	15.8×8.5	14.8×8.3	
***A. saghensis***									
CGM 40006 (holotype)^1^					9.5×5.5	12×5.9	10.7×4.7	8.5*×4.2	
***A. altidens***									
YPM 18160 (holotype)^1^					12.7×6.4	15.1×7.1		9.8*×5.7	
***A.*** ** cf. ** ***altidens***									
SMNS 12644^3^	28.2	13.1	15.1×8.4	13.3×8			13.1×7.1	9.4×6.3	
***A. gaudryi***									
MNHN unnumbered (holotype)^2^			14×?	14×9	11*×?	13×7.6			
HLMD unnumbered^2^			17.5×8.5	16.5×8.7	12*×?	16×9	13×9.5		
***A. intermedius***									
BSPG 2008 I 43 (holotype)^2^			14×8.2	13.2×7.3	12.2*×6*				
***Quasiapterodon minutus***									
SMNS unnumbered (holotype)^3^	16,2	8	?×4.1*	7.9×4.2	7.3×3.6				

**Abbreviations:** **c,** lower canine; **Dbm3,** depth beneath the third lower molar; **m,** lower molar; **p,** lower premolar; **Wbm3,** width beneath the third lower molar. Asterisks are for estimations. Data come from literature (^1,2^) and measurements taken by one of us (C.G.) based on casts or original specimens at the BSPG, NHM and SMNS (^3^).

1 Simons & Gingerich 1976.

2 Lange 1967.

3 This study.

**Table 2:** Measurements of upper dentition of Apterodontinae (in mm).

	M3	M2	M1	P4	P3	P2
***Apterodon langebadreae***						
BMNH M 85300 (holotype)	6.7*×16.1	14.8×14.8	10.7*×11.2*	12.7*×9.8*		
***A. macrognathus***						
BSPG 1905 XIII 9	?×18.3	17.3×18.9	11.7×13.1	16.8×13.5		
***A. altidens***						
SMNS 43465	10.4×13.3	13.4×16.4	13.5×13.3	14.1×12.2	14.3×9.1	12.7×8.3

**Abbreviations:** **M,** upper molar; **P,** upper premolar. Asterisks are for estimations. Measurements were taken by one of us (C.G.) based on original specimens at the BSPG, NHM and SMNS.

#### Differential diagnosis

Apterodontinae differing from *A. intermedius* and *A. gaudryi* by higher molar paraconids and a less broad m3 protoconid; from *A. gaudryi*, *A. altidens*, *A. macrognathus* by the presence of a m2 talonid; from *A. gaudryi* by a higher m2 hypoconulid; from *A. macrognathus* by the presence of a M3 metaconule; from *A. altidens* by unreduced metacone and metastyle on M2, a wider M3 on which is present a parastyle, a lower paracone, a more vestigial metacone and a wider protocone; from *A. saghensis* by a less important difference between p4 and p3 heigths.

#### Etymology

In honour and memory of Dr. Brigitte Lange-Badré, in recognition to her important contribution to the knowledge of the fossil hyaenodontidans.

#### Holotype

Partial skeleton belonging to one individual: BMNH M 85297, right mandible with broken c-m3; BMNH M 85298, left mandible with c, p2-m3, alveoli of i1–i3 and p1; BMNH M 85300, left maxilla with C-P1, P4-M3, alveoli of P2–P3; BMNH M 85301, fragment of right maxilla with broken I2-P2; BMNH M 85302, fragment of maxilla, BMNH M 85303, fragment of maxilla with P3; BMNH M 85304, skull fragment; BMNH M 85305, jaw/skull fragment; BMNH M 85306, skull fragment (frontal); BMNH M 85307, squamosal fragment; BMNH M 85308, squamosal fragment; BMNH M 85309, skull fragment (sagittal crest region); BMNH M 85310, skull fragment; BMNH M 85312, skull fragment (occipital region); BMNH M 85313, cervical vertebra; BMNH M 85315, left femur (lacking the most proximal end); BMNH M 85316 and 85317, distal and proximal right femur; BMNH M 85318, left humerus; BMNH M 85319, proximal right tibia; BMNH M 85320, distal right tibia; BMNH M 85321, distal right radius; BMNH M 85322, left ulna; BMNH M 85323, proximal right radius; BMNH M 85324, left metacarpal IV; BMNH M 85325, distal right metacarpal IV; BMNH M 85326, proximal right metacarpal IV; BMNH M 85327, phalange I; BMNH M 85328, phalange II; BMNH M 85329, phalange II; BMNH M 85330, rib; BMNH M 85331, distal right fibula; BMNH M 85332, fragment of left innominate; BMNH M 85333 fragment of right innominate; BMNH M 85334, fragment of right innominate; housed at the NHM.

#### Referred material

BMNH M 85335, cervical vertebra; housed at the NHM; DT24-12, broken lower molar; DT25-1, right mandible with broken p4-m2, alveoli of c, distal part of p2, p3 and m3; housed at the University of Al-Fateh, Tripoli.

#### Stratigraphic range and age

Localities 68.19 (type locality), 69.53 (see [36]), DT-Loc.24 and DT-Loc.25, Idam Unit [36], Dur At-Talah escarpment, Central Libya, Late Bartonian [33], [40].

#### General description

The holotype belongs to a subadult hyaenodontidan, as indicated by the fully erupted m3 and permanent premolars together with the unfused epiphyses of postcranial bones. Regarding the fossil preservation, elements of the right side have been more exposed to weathering than those of the left side.

#### Description of the mandible and lower dentition (Figures 2A–C, Table 1)

The best preserved mandible is the specimen BMNH M 85298. The ascending ramus is broken dorsally, behind the m3 alveolar level, and distally before the condyles. The horizontal ramus shows three mental foramina in lateral view: beneath the p1–p2 contact, the p3–p4 contact, and a very small one under the p3 distal root. On DT25-1, the last mental foramina is absent or fused with the second one. In medial view, the mandibular symphysis extends ventrodistally to end at the level of the p3 distal root. The mental foramen opens approximately 3.5 cm behind the m3, slightly beneath the alveolar level. The jaw is rather shallow and particularly long, measuring approximately 16 cm from the beginning of the tooth row to the most distal part of the remaining ascending ramus.

The dental formula is 3i, 1c, 4p, 3m. Except a small diastema visible between p2 and p3, the teeth are closely set together. The teeth morphology is more tubercular than sectorial as shown by the bulbous cusps. The incisors are so crowded that i1–i2 alveoli are located along a vertical axis. The canine is large and tends towards the labial side of the jaw. The p1 is single-rooted and more labially situated than the p2, as indicated by the alveolus. The other premolars of the jaw are two-rooted. The p2 and p3 are low-crowned and simply built: they bear a unique cusp, mesially placed with respect to the crown length, making the teeth asymmetrical in occlusal and lateral views. The p2 is more asymmetrical than the p3. Mesial and distal crests extend from the main cusps of these premolars to join the crown base. The p4 shows a tall main cusp, centrally positioned relative to the crown length, a tiny mesial shelf and a short and low talonid. The better preserved p4 on DT25-1 shows a talonid bearing an oblique cristid obliqua. Mesial and distal crests extend from the main cusp to join mesially the crown base and distally the cristid obliqua. The length and height of the premolars increase from p2 to p4. They are surrounded by a thick cingulid, more pronounced on the lingual side than on the labial one for BMNH M 85298 and at least well developed on the lingual side for the p4 of DT25-1 (worn labial side). The m1 is shorter than the p4, m2 and m3. It is broken and worn until the crown base, only the lingual side and the distolabial part of the talonid enamel being preserved. The trigonid outline is however visible. It seems to have retained a metaconid base and is slightly longer than the talonid. The poorly preserved m1 on DT25-1 shows also a shallow talonid basin. The m2 and m3 have a similar length. The trigonid is higher than the talonid. The paraconid, bending lingually, is the smallest cusp of the trigonid. The protoconid is the highest trigonid cusp. It is broader and distolabially situated relative to the paraconid. A faint enamel fold, which can be assumed as a vestigial metaconid, stands up on the distolingual side of the protoconid. This feature is visible on m3 but was certainly present on m2 also, the reduction of the metaconid being generally more important for the distal molars than for the mesial ones. The carnassial notch on the paracristid is short and feebly transversal, due to the weak lingual and labial extensions of the paraconid and the protoconid, respectively. A small precingulid rises just labial and basal to the paraconid. The paraconid position relative to the protoconid is more lingual in m3 than in m2. This makes the paracristid more transversally oriented in m3. Moreover, compared to the m2, the m3 paraconid and protoconid are higher and the precingulid is more developed. The talonid breadth is similar to the trigonid one in m2 but smaller in m3. Parallel to the labial border of these two molars, the talonid bears a short and weakly oblique cristid obliqua, extending distally from the base of the protoconid distal face to join a prominent and bulbous hypoconid. The hypoconid and the protoconid are aligned mesiodistally in m2. The hypoconid in m3 is slightly more lingual than the protoconid. In both molars, the labial cingulid stands up abruptely on the distal talonid, merging with the hypoconulid. The m2 hypoconulid is slightly lower than the hypoconid and separated from it by a narrow posthypocristid. These cusps are worn in m3. The m2 talonid also bears a shallow basin, absent in m3. Both molars have a crestiform entoconid surrounding lingually the talonid and merging mesially with the lingual cingulid.

#### Description of the upper dentition (Figure 2D–E, Table 2)

The upper dentition is represented by several maxilla fragments, BMNH M 85300 showing the best preserved dental crowns: the distal part of the canine, the P1, the P4 enamel outline and labial wall, the M1 lingual and distolabial parts, and the M2–M3. BMNH M 85301 preserved the roots of I2-P2. BMNH M 85303 is a fragment of maxilla bearing a P3.

The teeth are situated closely to one another, except between the canine and the third incisor. The canine is a large and slightly curved tooth. The P1–P3 are double-rooted. The P1 and P2 have probably shown similar morphologies compared to the P3, which bears a single cusp from which two crests extend mesially and distally to join the cingulum at the crown base. Judging from the alveoli length, the P3 was longer than the P2 and even more than the P1. The P4 and the molars are three-rooted. The P4 is the most complex premolar. It bears a prominent conical paracone and a small protocone lobe (i.e., without any individualized cusp). A distinct parastyle is visible at the mesial base of the paracone. On the distolabial corner of the tooth, a marked metastyle gets in contact with the mesiolingual base of the M1 paracone. The P4 has been probably shorter but wider than the P3. The molars show well-developed cingula. The M1 and M2 are subtriangular in shape and tritubercular. The M1 is shorter and narrower than the M2 but has probably shown a similar general morphology. The paracone and metacone are closely attached, fused at their base. The paracone and the metacone, which is weakly more lingual, have approximately the same heights and widths. Relative proportions of these labial cusps could have been slightly different for the M1. The metastyle is short and distolabially oriented. A shallow ectoflexus lies labially to the paracone-metacone contact. The ectoflexus might have been more marked in M2 than in M1, because the metastyle, which bears a distinct carnassial notch, is longer and oriented more distolabially in M2. The M1 metastyle is more labially positioned than the P4 one, as it is in contact with the M2 parastyle region, just mesiolabially to the M2 paracone base. The M2 metastyle reaches the M3 parastyle level. A small parastyle is visible at the mesiolabial base of the M2 paracone. This part is broken in M1. The protocone is prominent and mesially oriented, due to the oblique postprotocrista. It is lower than the labial cusps. The protocone of M2 is also displaced more lingually than in P4 and M1. Small para- and metaconules are present at the base of the M2 paracone and metacone, respectively. The paraconule is more developed than the metaconule. Their presence can not be inferred for M1. The M3 is transversally developed, wider but shorter than M2. It bears a strong parastyle, as high as the paracone. The paracone is centrally placed with respect to the longitudinal axis of the tooth. It is lower than the M2 paracone. A tiny metacone is retained, but is nearly entirely fused with the distal face of the paracone. A metaconule is present just lingual to this metacone and is nearly as developed as the M2 paraconule. The mesial corner of the M3 being damaged, the presence or the lack of a paraconule is not observable. The M3 protocone is lower than in M2. Its base extends more lingually than in the preceding molars and its tip is more labial and does not project mesially.

#### Description of the basicranium and postcranium (Figures 3A–C and 4A–D, Table S1)

On the basicranial fragment BMNH M 85312, the foramen magnum is transversally wide and the occipital appears fan-shaped in distal view, as in *Apterodon macrognathus*. The most ventral part shows two scars which are probably the insertion sites of the *M. rectus capitis posterior minor*. The axial skeleton is represented by several vertebrae and a rib. Fragments of two cervicals are preserved. BMNH M 85335 shows a dorsally broken spinous process. Ventral to this process, zygapophysis processes are present laterally and medially. The spinal canal is wide and the underneath vertebral body (centrum) is ovoid in proximal view and triangular-shaped in distal one. The more ventral transverse processes are thin, oriented ventrolaterally and ventromedially, and show small transversarium foramina at their base. The specimen BMNH M 85335 seems to have a more distal position on the vertebral column compared to BMNH M 85313 as indicated by its wider spinal canal and its more mesially inclined spinous process. Both vertebrae can be c3–c6 as the c7 misses transversarium foramina and as the c1–c2 lacks a centrum and presents an odontoid process, respectively. The rib (BMNH M 85330) shows two articular surfaces with a vertebra: a dorsal tuberculum, which articulates with the transverse process, and a ventral capitulum, which meets on adjacent vertebral centra. As both structures are present and well-separated on this bone, it belongs probably to the proximal thoracic ribs.

The forelimb (Figures 3A–C, Table S1) is represented by a humerus, an ulna, a radius and two metacarpal bones. The humerus (BMNH M 85318; Figure 3A) is a complete left bone with minimal damage on the regions of the lateral supinator crest and the medial epicondyle. The bone is relatively short and robust (humeral width to length = 32%, Index 1 in Table S1; cf. functional interpretation). The humeral head is large, laterally compressed and appears convex in lateral and medial views, the neck bowing moderately ventrally. The lesser tuberosity, where an important medial rotator muscle of the shoulder inserts (i.e., *M. subscapularis*), is tightly pressed against the dorsomedial surface of the humeral head. Dorsolaterally to and slightly above this head rises the greater tuberosity for the attachment of muscles involved in the lateral rotation of the shoulder and abduction initiation (i.e., *M. teres minor*, *M. supraspinatus*, and especially *M. infraspinatus* for which a prominent pit is present on the ventrolateral border of the tuberosity). In proximal view, the greater tuberosity has an oblique direction relative to the dorsoventral axis. It extends dorsally, delineating with the lesser tuberosity a wide and shallow bicipital groove for the tendon of *M. biceps brachii*, involved in the supination and flexion of the elbow. The deltopectoral crest, where important muscles for abduction, adduction, extension and medial rotation of the forelimb insert, rises above the humeral shaft. It extends dorsally and distally from the greater tuberosity to about three-fourths down of the bone, and tapers to merge with the diaphysis. The distal part of the humerus is divided into a curved trochlea for articulation with the ulna and a slightly convex capitulum for articulation with the radius. Moreover, the medial rim of the trochlea is more distal than the capitulum and the medial epicondyle. In ventral view, a supratrochlear foramen and a deep fossa are visible proximal to the trochlea. The distal humerus shows a strong medial epicondyle protruding ventrally, maybe related to the presence of an entepicondylar foramen [64]. From this epicondyle originate the *M. pronator teres* as well as carpal and digital flexors. The broken proximal part of the medial epicondyle prevents to check the presence of an entepicondylar foramen. The lateral epicondyle is relatively short. In lateral view, it bears a prominent scar which is probably the insertion site for the common extensor tendon, origin of the digital and carpal extensors. A relatively developed lateral supinator crest extends proximoventrally to merge with the diaphysis about one-thirds the length of the bone.

BMNH M 85322 is an entire left ulna (Figure 3B). It is slightly shorter than the humerus (Table S1). The diaphysis is slightly compressed lateromedially and somewhat curved in lateral view. The proximal olecranon, where the major extensor of the forelimb (i.e., *M. triceps*) inserts, does not bend dorsally or ventrally with respect to the longitudinal axis of the bone, but is slightly medially inclined. It is long relative to the entire length of the ulna (approximately as long as the semilunar notch), as found generally in hyaenodontidans [65] (olecranon to ulnar length = 22%, Index 2 in Table S1; cf. functional interpretation). Below the olecranon, the dorsal semilunar notch for the distal humerus is concave. Immediately distal to the lateral part of this notch is a relatively flat radial notch for the contact with the proximal radius. This notch is elongated dorsomedially to ventrolaterally but somewhat more dorsally directed. In dorsal view, the flange of the anconeal process, proximal to the semilunar notch, is concave and asymmetrical. Its medial rim is more proximal than the lateral one. A deep and longitudinal groove for the *M. abductor pollicis longus* (abductor of the thumb at the carpometacarpal joint and partial abductor of the wrist) is present along the lateral side of the ulna. Just lateral to the semilunar notch, this groove is delineated by a strong ventral ridge, where might be inserted the *M. flexor digitorum profundus*, a powerful digital flexor. A thinner ridge, more dorsally located and immediately distal to the semilunar notch, contributes to delineate the dorsal side of the groove. These ridges taper distally to disappear at the two-thirds of the bone, at the level of the interosseous tubercle which projects laterally. On the dorsodistal surface of the ulna, the region for the *M. pronator quadratus*, the main forearm pronator, is partly broken. The distal ulna presents a small radial facet for articulation with the ulnar facet of the radius, and a more distal and ventral massive styloid process for the articulation with the carpal.

BMNHN M 85323 (Figure 3C) and BMNH M 85321 are damaged proximal and distal ends of a right radius, respectively. Both bones were probably from the same radius. The proximal part of the diaphysis is compressed dorsoventrally. Thus, the radial head has an elliptical outline, and its ventral margin is weakly convex for the articulation with the proximal ulna. The radial head, which also articulates with the distal humerus, shows a feebly concave proximal facet and appears sigmoid in dorsal view because of a high capitular eminence. The bicipital tuberosity region is weathered. The distal part of the radius (BMNH M 85321) is badly preserved. It has a squared overall shape in cross section. The facets for the carpals (scaphoid and lunate) are indistinct. Only the lateral styloid apophysis is recognizable.

The only metacarpal bones preserved are two metacarpals IV: a left (BMNH M 85324) and a right one (BMNH M 85325 and 85326). BMNH M 85324 shows a proximal epiphysis with a convex articular facet for the unciform. On the medial face, the articular facet for metacarpal III forms an oblique crest, dorsodistally extended. The lateral articular facet for metacarpal V extends proximodistally along the proximal and dorsal borders of the bone. The distal part of the diaphysis and the distal epiphysis bow slightly ventrally.

The hindlimb (Figure 4A–D) comprises two innominate bones, three femur specimens, one tibia and one fibula. Right and left innominate fragments are preserved. The left portion (BMNH M 85332; Figure 4A) is the less damaged one. It lacks the complete pubis and ilium. The ilium is broken proximally, just in front of the anterior inferior iliac spine. This spine, close to the acetabulum, has a very distal position. It is marked by a deep pit visible in dorsal view. The ilium neck is short and broader than the proximal branches of the pubis and the ischium. In lateral view, the acetabulum appears circular and is delineated proximally and dorsally by a high rim. Its lunate articular surface opens distoventrally. A broad rugosity, where inserts the *M. rectus femoris*, a powerful hip flexor and knee extensor, lies immediately proximal to the acetabulum. Ventrally to the acetabulum, a broad tuberosity for the *M. iliopsoas*, involved in flexion and lateral rotation at the hip joint, is present. The ramus of the ischium is relatively flat lateromedially. The acetabulum and the descending ramus of the ischium face more laterally than ventrally, as the descending ramus is not deflected outwards with respect to the ilium. Distal and dorsal to the acetabulum, the ischial spine, usually bearing the *M. gemelli* (lateral rotator and hip abductor), is poorly convex. Even if the pubis and the ischium are not entirely preserved, the obturator foramen, distal to the acetabulum, has been probably broad and elongated proximodistally.

Three specimens of femur (Figure 4B) are attributable to *A. langebadreae* nov. sp. BMNH M 85315 is a subcomplete left bone, lacking its femoral head, and BMNH M 85317 and 85316 are proximal and distal epiphyses, respectively, of a right femur. The femur is longer than the humerus (Table S1). It is compressed dorsoventrally and its proximal part is twisted medially. On the specimen BMNH M 85317, the head, which articulates with the acetabulum, is nearly round and does not extend on the femoral neck. The neck is relatively long, so that the femoral head is bowed medially. In medial view, the fovea capitis, an ovoid depression where inserts the *ligamentum teres*, is located ventrally. Laterally to the femoral head lies a roughened greater trochanter for the attachment of the *M. gluteus medius*, *M. gluteus profundus* and *M. piriformis*, powerful hip extensors. It stands below the level of the femoral head. Distal to the proximal end of the femur are the lesser and third trochanters (on BMNH M 85315 and 85317). Below the femoral head, the prominent medial lesser trochanter, where the *M. iliopsoas* inserts, projects more medially than ventrally. Between the lesser and greater trochanters lies on BMNH M 85315 a deep trochanteric fossa, the attachment area of several lateral hip rotators. The trochanteric line, bordering laterally this fossa, is very thick. The lateral third trochanter, distal to the greater trochanter, is well-developed and slightly ventral. It is the insertion area of the *M. gluteus superficialis*. The distal femur bears a medial condyle more ventrally developed than the lateral one. In distal view, the patellar trochlea is weakly grooved and appears slightly more extended dorsoventrally than wide mediolaterally. The intercondylar fossa is as deep dorsoventrally as wide mediolaterally. The medial epicondyle bears a deep central depression, where may have been attached the medial collateral ligament, and a smaller pit, just proximal to it. This last area could be the attachment site of the medial head of the *M. gastrocnemius*, involved in plantar and knee flexions. The lateral epicondyle is rugose and bears distinct pits, certainly related to the insertion of tendons such as that of *M. popliteus*, which provides the flexion of the leg upon the thigh and unlocks the knee by rotating the tibia inward. They also might contain the insertion site of the lateral collateral ligament.

BMNH M 85319 and 85320 are two damaged proximal and distal ends of a right tibia (Figure 4C). The condyles are partly broken (especially the medial one) and weathered. The dorsal tibial tuberosity is short and extends distolaterally in a drop shape. This tuberosity is not projected far dorsally and has a blunt outline. It constitutes the dorsalmost margin of a concave lateral surface on the proximal tibia, where inserts the *M. tibialis anterior* (responsible for the dorsiflexion and the inversion of the foot). The distal tibia is badly preserved, only the medial edge of the medial malleolus and the ventrolateral edge of the talar articular facet are visible. The tubercule for the *M. tibialis posterior* tendon is parallel to the ventral border of the medial malleolus.

BMNH M 85331 is a distal end of a right fibula (Figure 4D). The contact facet with the tibia is unidentified regarding the poor preservation state of the distal tibia. Two facets with the tarsal bones are visible on the lateral malleolus: the more lateral and distal one is in contact with the dorsal facet of the calcaneus, and the more medial and proximal one articulates with the lateral facet of the astragalus. The calcaneal facet of the fibula has a sinuous lateral edge and is dorsoventrally oblique. The astragalar facet is nearly oriented in the proximodistal axis of the bone. The separation between the two facets is roughly defined, except near the ventral crest which joins the lateral edge of both surfaces. Together with a substantial lateral protuberance, located proximally and laterally relative to the lateral malleolus, the ventral crest draws a large concave surface, analogous to the structure developed in other hyaenodontidans (i.e., *Sinopa grangeri* [66]; pp. 227). This results in an arrowhead shape of the epiphysis in distal view.

Other limb bones are preserved: one first (BMNH M 85327) and two second phalanges (BMNH M 85328 and 85329). The second ones are twice less long than the first phalange. They are flattened dorsoventrally and are shorter than the metacarpals. An association with a hand or a foot is not possible.

#### Comparisons and discussion

*Apterodon* species are mostly represented by dental material, except *A. macrognathus* for which postcranial bones are also reported [12], [16].

Two species of *Apterodon* are known from the early Oligocene of Europe. *A. gaudryi* is known from the Quercy fissures of France (probably of early Oligocene age) and from Flonheim, in the german Mainz Basin [31], [32], [59]. This species is represented by two mandibles which differ from the material of *A. langebadreae* nov. sp. in bearing lower paraconids on molars, a lower m2 hypoconulid, a broader m3 protoconid, and in lacking a m2 talonid basin. Molars from the French specimen of this species have roughly the same proportions as the holotype of *A. langebadreae* nov. sp. (Table 1). The mandible of *Apterodon intermedius*, the second European species discovered in the german Weisselter Basin (locality of Espenhain, MP22), was described by Lange-Badré & Böhme [30] as bearing p4-m2. However, the dimensions of the first tooth (shorter than the presumed m1–m2) and the morphology of the last molar (short and narrow talonid, very lingual paraconid, similarly placed as in m3 of *Apterodon* species, and trigonid similar in shape to the m3 of *A. gaudryi*) rather suggest a m1–m3 dental succession and a close morphological similarity with *A. gaudryi*. *A. intermedius* differs from the specimens of *A. langebadreae* nov. sp. by lower paraconids and a broader protoconid on m3.

Three species of *Apterodon* are described from the Eocene-Oligocene Fayum depression of Egypt. *A. macrognathus* (the best represented *Apterodon* species) and *A. altidens* are known from the late Eocene-early Oligocene beds of the Jebel Qatrani Formation [12]–[14], [16]. The oldest egyptian species, *A. saghensis*, is recovered from the late Eocene Qasr el-Sagha Formation [61]. *A. langebadreae* nov. sp. exhibits smaller proportions compared to *A. macrognathus* (Tables 1–2 and [12], [16]). The new Lybian species differs from it by the presence of a talonid basin on m2 and a M3 metaconule. Concerning the postcranial remains, the humerus of *A. langebadreae* nov. sp. differs from that of *A. macrognathus* (BMNH M 9257, cast of CGM 9445; figured by Andrews [12], pp. 229) in having a more developed lateral epicondyle and a stronger dorsoventral shortening of the distal part of the diaphysis relative to the proximal one. The flange of the anconeal process of the ulna is also less elongated proximomedially and the ventral ridge of the scar for the *M. abductor pollicis longus* is more dorsally placed than in the broken ulna of *A. macrognathus* (unnumbered specimen from SMNS; described in Schlosser [16], pp. 80). The radial head is more curved in dorsal view with a higher capitular eminence by comparison to the complete radius SMNS 43467 attributed to *A. macrognathus* (described in Schlosser [16], pp. 80). Finally, no significant differences are observable relative to the fragmentary tibia of the Egyptian species (unnumbered specimen from SMNS; described by Schlosser [16], pp. 80–81). *A. langebadreae* nov. sp. differs also from *A. altidens* by the presence of a m2 talonid basin and by an unreduced metacone and metastyle on M2, a wider M3 (Table 2) on which is present a parastyle, a lower paracone, a wider protocone, and a more vestigial metacone. The third Fayum species, *A. saghensis*, is represented by a mandible only bearing p2–p4 and the basal crown of m1. This mandible shows smaller teeth compared to *A. langebadreae* nov. sp. (Table 1) and the p4 is more than half higher than p3. The height difference between these premolars is much more important than in *A. langebadreae* nov. sp., although this character is likely to be variable within species [67]. However, the attribution of the Qasr el-Sagha mandible to a separate species relative to the two other Fayum taxa is also questionable, premolars of *Apterodon* bearing very few characters to be diagnostic at the species level.

Another occurrence of *Apterodon* has been recently reported in Eastern Africa. The isolated tooth (KNM-LH 10249) of *Apterodon* sp. from the late Oligocene of Lokone, in Kenya [20], is probably a m2, judging from its relatively mesial paraconid position and its talonid size and morphology. The m2 of *A. langebadreae* nov. sp. differs from it in being wider and in having a higher paraconid and a talonid basin.

**Figure 5:**
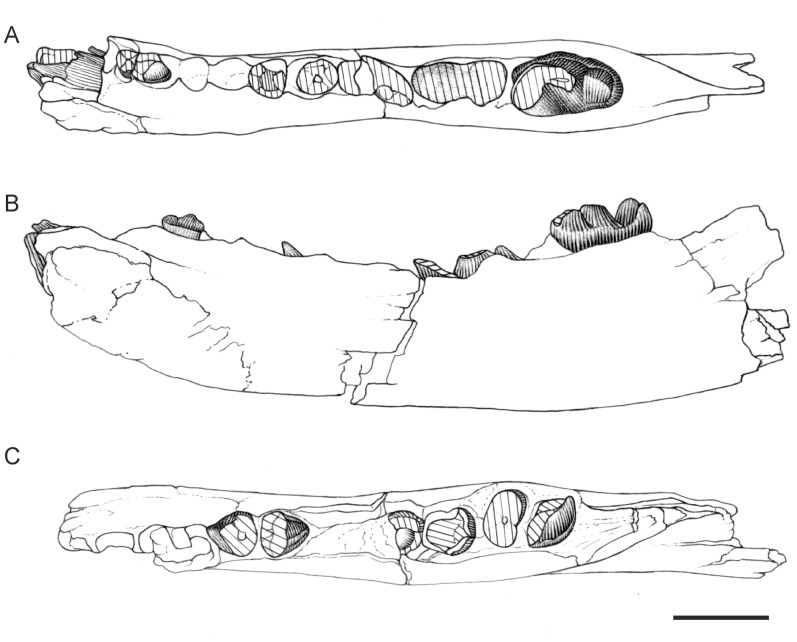
Dental material of *Apterodon* indet. DT24-1, right mandible with canine base, m3 and roots of p1-m2, in occlusal (A) and lingual (B) views; DT24-2, left mandible with broken p4 and m3, roots of p2–p3 and m1–m2, in occlusal view (C). Scale = 1 cm. Drawings by Sabine Riffaut.

*Apterodon* indet.

(Figures 3, 4, 5, Table S1)

#### Material

DT23-1, right mandible with heavily worn p2-m3; DT23-2, left mandible with well-damaged m2–m3; DT24-1, right mandible with a broken m3, p1-m2 roots and the canine base; DT24-2, left mandible with broken p4 and m3, roots of p2–p3 and m1–m2; DT24-3, left calcaneus; DT24-4, right calcaneus; DT24-5, right astragalus; DT24-6, right femur; DT24-8, caudal vertebra; DT24-9, distal femur (with condyles); DT24-10, basicranial element (with condyles); DT24-11, caudal vertebra; DT24-13, distal left humerus; DT24-14, proximal right radius; housed at the University of Al-Fateh, Tripoli.

#### Stratigraphic range and age

DT-Loc.23 and DT-Loc.24, Idam Unit [36], Dur At-Talah escarpment, Central Libya, Late Bartonian [33], [40].

#### Description of the mandibles and teeth (Figures 5A–C, Table 1)

Specimens DT24-1 and DT24-2 correspond to a unique individual. The mandibles are broken at the beginning of the ascending ramus and the left mandible is damaged laterally ahead of the p4. They show two mental foramina: one below the p4 mesial root and another at the canine base. The symphysis extends distally underneath the p3–p4 contact. Most teeth are broken at the base of the crown. However, the m3 of DT24-1 and the p4 and m3 of DT24-2 are partially preserved. The teeth have certainly been closely set together and double-rooted, except the p1, as indicated by the alveoli. The alveolar length increases from p1 to p4 and m1 was shorter than m2 and m3, both having approximately the same length. The p4 bears a tiny mesial cusp and small talonid with a sharp and oblique cristid obliqua, lower and more labial than on m3. The distal p4 talonid is surrounded by a cingulid. The m3 trigonid lacks metaconid and bears a paraconid lingually placed with respect to the protoconid. The trigonid cusps are higher than the talonid. A strong precingulid lies mesiolabially. The talonid, shorter and narrower than the trigonid, shows a strong cristid obliqua, distolingually oriented relative to the tooth row, and a crestiform hypoconid, centrally positioned relative to the talonid width. Lingual to the cristid obliqua and hypoconid, the talonid shows a concave face. The cingulid surrounding the tooth appears thicker at the level of the talonid, where it arises just distal to the crestiform hypoconid and is located lower to it. On DT23-1, two mental foramina are visible, below the p4 mesial root and the p2 distal root. The position of the foramina is variable in at least one species of the genus, *A. macrognathus*, as observed by one of us (C.G.) in the collections of the AMNH, BSPG, NHM and SMNS. The size of the mandible and the molar morphology of DT23-1 are similar to those of DT24-1 and DT24-2.

#### Description of the postcranium (Figures 3D–E and 4E–G, Table S1)

DT24-13 is a distal left humerus (Figure 3D). The end of the deltopectoral crest is visible in dorsal view. The distalmost parts of the medial epicondyle, the medial rim of the trochlea and the capitulum are at the same level. The medial epicondyle is well-developed relative to the mediolateral width of the distal humerus. This involves powerful carpal and digital extensors. The medial epicondyle also bears an entepicondylar foramen, elongated in an oblique direction. A small supratrochlear foramen opens proximally to the trochlea and capitulum. The supinator crest is well-developed and laterally projected.

DT24-14 is a proximal right radius (Figure 3E). The articular surface for the capitulum is not entirely preserved as the ventrolateral part of the head is broken. This surface is elliptical and sigmoid. In lateral view, the diaphysis is curve and flares below the bicipital tuberosity. Slightly lateral to the bicipital tuberosity and below it, is a deep scar possibly for the insertion of the *M. flexor profundus digitorum*.

DT24-6 is a right femur (Figure 4E). The femoral head is nearly round. The neck is short. In medial view, the fovea capitis is elongated from the centre of the head to its distoventral corner. The trochanteric fossa is very deep. The trochanteric line, bordering laterally this fossa, is very thick. The greater trochanter projects slightly above the femoral head. The lesser trochanter is somewhat ventrally oriented, certainly due to the twisted proximal epiphysis. The third trochanter is lateral and feebly developed. The medial condyle of the distal femur is more ventrally developed than the lateral one. The patellar trochlea is well-grooved. The distal femur shows similar medial and lateral depressions as in *A. langebadreae* nov. sp.

DT24-4 (right specimen) is the most complete calcaneus (Figure 4F). The proximal part of the calcaneus presents a well-developed medial tubercle. The calcaneal tuber lacks any dorsoventral groove between the medial and lateral tubercles. In dorsal view, the distal end of the tuber shows a small depression in front of which occurs the fibular facet, a convex surface extended distolaterally. Along its medial side is a slightly longer and wider ectal facet for the articulation with the lateral portion of the ventral astragalus. This ectal facet is sinuous and convex. On the medial side of the calcaneus lies the *sustentaculum tali*. In dorsal view, it appears as a concave and nearly squared surface for articulation with the sustentacular facet of the astragalus. Along the medial side of this facet is a thin tibial facet that extends distally to the *sustentaculum tali* until nearly contacting the cuboid facet. On the ventral face of the *sustentaculum tali* is a marked groove for the tendon of the *M. flexor digitorum longus*. The cuboid facet is weakly concave, oval and dorsolaterally directed. The dorsal edge of this facet is more distal than the ventral one. Adjacent to the lateral edge of the cuboid facet, the peroneal tubercle, where inserts notably the *M. peroneus longus* for evertion, abduction and plantarflexion of the pes [68], might have been poorly developed.

A complete right astragalus is preserved (DT24-5; Figure 4G). The astragalar body shows an astragalar foramen proximally positioned. The dorsal trochlea, for articulation with the distal tibia, is slightly grooved. In distal view, the lateral margin appears slightly higher than the medial one. The medial margin ends more proximally and ventrally, beyond the astragalar foramen. Two articulation surfaces for the calcaneus, the sustentacular and the ectal facets, are present in the ventral side of the astragalar body. They constitute the subtalar joint. The sustentacular facet, for articulation with the medial facet of the calcaneus, is slightly convex and shows a D-shape, the more lateral border being curved inwards. The ectal facet, for articulation with one of the lateral facet of the calcaneus, is elongated distolaterally, shallow, and almost entirely flat. Its proximal border is, however, concave and visible in lateral view. It is separated from the lateral fibular facet by a small and shallow groove, until joining it in its distal end. The sustentacular and ectal facets are separated by a deep and wide groove, the astragalar sulcus, where insert the tendons of plantar flexors. The sulcus leads at its proximal end to the astragalar foramen. In its medial side, the astragalar body bears a strong proximal protuberance. The neck is short proximodistally and wide lateromedially. The head is constituted by a convex navicular facet extending slightly medioventrally, as the long axis of the head is rotated about 20 degrees from the mediolateral plane. The medial border of this surface is nearly in contact with the distomedial part of the sustentacular facet. A small and less convex cuboid facet is present laterally to the navicular facet.

The dimensions of the proximal and distal caudal vertebrae (DT24-8 and DT24-11) could suggest the presence of a long tail. The proximal caudal vertebra, which bears broken transverse processes and a small spinal canal, is flattened ventrally.

#### Comparisons and comments

The lack of metaconid, the lack of talonid basin and the strong cingulid, notably distal to the hypoconid, remind m3 of *Apterodon* species. The dental and postcranial material is smaller than that of *A. langebadreae* nov. sp. (Table 1, Table S1). The overall shape of the postcranial bones strongly recalls that observed in *A. macrognathus* and *A. langebadreae* nov. sp. Some morphological differences compared to the new Libyan species are the broader medial epicondyle of the humerus, the more laterally extended supinator crest, the similar distal development of the medial epicondyle and medial rims of the capitulum and trochlea, the shorter femoral neck and thus the slightly higher greater trochanter, the more twisted proximal part of the femur, resulting in a more ventral lesser trochanter and a more lateral third trochanter, the less prominent third trochanter, and the narrower patellar groove. The proximal part of the radius is less fragmentary than that of *A. langebadreae* nov. sp. and shows a deep scar probably for the *M. flexor digitorum profundus* as well as a general curvature and distal enlargement of the diaphysis in lateral view, recalling the radius of *A. macrognathus* [16]. The calcaneus is roughly similar to that of *A. macrognathus* (BMNH M 8441A and BMNH M 8512; described by Andrews [12], pp.232) but has smaller and less wide proportions, like the astragalus. The astragalus differs from that of *A. macrognathus* (BMNH M 9259, cast of CGM 8116 and BMNH M 8441; figured by Andrews [12], pp.231) in having a slightly deeper trochlea due to a higher lateral margin relative to the medial one, and a shallow and shorter groove separating the ectal and fibular facet. We suspect that this material could belong to a new species. However, regarding the poorly understanding intraspecific morphological variation whithin *Apterodon*, we attribute these specimens to *Apterodon* indet. until additional dental material is found.

### Functional interpretation

#### Postcranium of *Apterodon langebadreae* nov. sp

The proximal humerus bears a greater tuberosity protruding beyond the articular head. This feature is found in cursorial and terrestrial carnivorans (e.g., *Canis*, *Panthera, Civettictis*, *Herpestes, Ichneumia*), but also in semi-fossorial (*Taxidea, Meles*) and semi-aquatic ones (*Lutra*) [69]–[71]. The deltopectoral crest is long, which suggests a mobile shoulder and powerful flexion and extension of the forelimb, like in carnivorans which show manipulative behaviour and which can dig, swim or climb by bracing [71], [72].

The trochlea morphology of the humerus of *A. langebadreae* nov. sp., which medial rim is distal and angled with respect to the capitulum, limits movements in a parasagittal plane, although to a lesser degree compared to cursorial and fossorial forms such as *Civettictis*, *Canis* and *Meles* [69], [71]. The perforated and deep supratrochlear fossa of the Libyan apterodontine allows a great extension of the elbow joint [68]. The olecranon is long relative to the longitudinal length of the ulna (e.g., see olecranon to ulnar length in [73]), as it tends to be in fossorial and aquatic mammals by opposition to cursorial forms [74]. This suggests a more efficient moment arm for notably the *M. triceps*, facilitating a more powerful extension of the forelimb during digging and swimming strokes. In hyaenodontidans and carnivorans, the ulna grows ventroproximally during ontogeny [65]. Thus, adult individuals of *A. langebadreae* nov. sp. should have a more ventrally projected olecranon than subadult ones. Although it is difficult to know to what extent the projection of the ulna was ventral, like it is in extant terrestrial trotters and cursorial carnivorans (e.g., *Ichneumia*, *Canis*), it is therefore different from scansorial or arboreal taxa (e.g., *Potos*, *Arctictis*, *Paradoxurus*) for which the olecranon projects clearly dorsally, resulting in a flexed elbow (see [75] for comparative olecranon angle measurements). The scansorial and arboreal mammals also exhibit a substantial mobility of the radius allowing a great range of pronation and supination. On the contrary, in *A. langebadreae* nov. sp., the nearly flat and dorsally positioned ulnar facet for the radius demonstrates a stable radioulnar joint. The elliptical shape of the proximal radius and the high capitular eminence involve a stable humeroradial joint. These features reduce abduction-adduction and supination capability compared to climbers [76]. It is worth mentioning that aquatic mammals tend to restrict rotation of the forearm (manus held about mid-way between full pronation and full supination) [77] as well as fossorial taxa, although a certain degree of pronation/supination ability is necessary to manipulate food and to dig [74].

On the distal ulna, the insertion site for the *M. pronator quadratus*, usually well-developed in climbers (e.g., *Potos* [78]), does not seem to have been prominent in *A. langebadreae* nov. sp. The wide medial epicondyle of the humerus of the Libyan species traduces the insertion of powerful flexors for the manus and pronator, like in semi-fossorial (*Meles*, *Mungos*, *Taxidea*), arboreal (*Nandinia*, *Arctictis*) and semi-aquatic (*Lutra*, *Enhydra*) carnivorans [69], [70]. The development of a wide lateral supinator crest is particularly important for fossorial (*Meles*, *Taxidea*) and aquatic (e.g., *Enhydra*) adaptations. The lateral scar on the ulna for the *M. abductor pollicis longus* is very deep and the insertion ridge for the *M. flexor digitorum profundus* is very well-marked, thus suggesting important mobility of the manus (strong abduction of the wrist and the thumb and powerful flexion of the digits), as expected in fossorial and aquatic mammals.

Concerning the forelimb morphology, the new Libyan species shows a lower range of mobility of the elbow joint compared to scansorial and arboreal mammals (angled medial rim of the trochlea, elliptical humeral articular facet of the radius and high capitular eminence, nearly flat and dorsal radial facet on the ulna), thus reducing supination capacity. It shows flexible wrist and digits (strong medial epicondyle on the humerus, lateral scar for the *M. abductor pollicis longus* and strong crest for the *M. flexor digitorum profundus* on the ulna), as well as powerful extension and flexion of the forearm (long deltopectoral crest, deep and perforated supracondylar fossa, and strong lateral crest on the humerus, long olecranon on the ulna), useful for diggers and swimmers. The humeral shaft of *Apterodon langebadreae* nov. sp. is strongly curved in lateral view and strongly flares dorsoventrally in its proximal part, recalling the morphology of aquatic mammals. Although hyaenodontidan limbs are usually short with respect to body size in comparison to carnivorans [79], the shorthening of the humerus compared to the radius and ulna (Table S1) is characteristic of aquatic and fossorial adaptations (see brachial index in Davis [80], pp.35–37 and Gebo & Rose [81], pp.130), providing a powerful extension of the forearm. The mediolateral compression and dorsoventral thickness of the humeral shaft is also typical of swimmers [82].

The orientation of the ischium and the acetabulum with respect to the ilium suggests an abducted position of the femur. In fact, the more angled is the pelvic floor (i.e., acetabulum and ischium face laterally rather than ventrally), the more abducted is the femur [83]. This angle is more pronounced in *A. langebadreae* nov. sp. compared to cursorial carnivorans (e.g., *Panthera*, *Canis*) and to a lesser extent relative to *Ursus* [71]. Moreover, the fovea capitis of the femur is not centered in medial view, unlike in cursorial carnivorans such as *Felis* and *Canis* [83]. The hip joint shows a usual flexed posture, as indicated by the broad rugosities of the innominate for the *M. rectus femoris* and *M. iliopsoas*, huge hip flexors. The ischial spine of the innominate, usually well-developed in climbers such as *Potos* and *Arctictis* [68], is feebly visible in *Apterodon langebadreae* nov. sp. This condition recalls that observable in semi-fossorial (*Meles*) and aquatic (*Lutra*, *Phoca*) carnivorans. On the femur, a medial lesser trochanter, as found in scansorial and arboreal taxa, is suggested to enhance the capacity of lateral rotation at the hip, whereas a more ventrally projected lesser trochanter, as found in *A. langebadreae* nov. sp. and in terrestrial and cursorial carnivorans (e.g., *Viverra*, *Ichneumia*, *Herpestes*) is supposed to provide less lateral rotational mobility of the hip [84]. The third trochanter is prominent and could traduce a powerful *M. gluteus superficialis* responsible for the flexion and abduction of the femur. The greater trochanter of the Libyan species is located below the femoral head, unlike in cursorial specialists (e.g., *Panthera*, *Canis*), and therefore resembling that of arboreal and terrestrial taxa (e.g., *Potos*, *Ursus*). This feature suggests a less powerful extension of the femur compared to runners.

The distal femur shows a medial condyle longer than the lateral one, so that the lateral condyle reaches the end of its travel before full extension. The femur then rotates medially which renders the knee joint stable [85]. The medially-twisted shaft of the femur also contributes to stabilize the knee joint. The distal trochlea is more grooved and narrower in *Apterodon langebadreae* nov. sp. compared to *Ursus* and *Paradoxurus*. This could suggest a more parasaggital excursion of the leg relative to the thigh, as notably found in cursorial mammals [71]. However, the asymmetrical condyles indicate at least slight rotation capabilities of the leg relative to the thigh. The development of pits on the medial and lateral epicondyles, notably those for the *M. gastrocnemius* and the *M. popliteus*, traduces a flexed knee posture and a stable knee joint. Dorsally, the tibial tuberosity is relatively close to the proximal condyles, a tendency seen in the terrestrial *Ursus*, the semi-fossorial *Meles* and the semi-aquatic *Lutra*, by opposition to the cursorials *Canis* and *Panthera*, for which the tuberosity is more ventral.

Concerning the hindlimb morphology, the very distal position of the sacral articulation of the innominate could suggest a rather short ilium, as it tends to be in aquatic mammals [82]. In Carnivora, pinnipeds have a shorter ilium compared to the ischiopubis whereas their lengths are similar in otters. Swimmers also tend to have a shorter femur relative to the tibia and fibula (see crural index in Davis [80], pp.35–37), which is unfortunately not measurable in *A. langebadreae* nov. sp. due to the broken crus. The robust and dorsoventrally compressed femur however recalls the morphology of otters. The hip joint shows a usual flexed posture (powerful *M. rectus femoris* and *M. iliopsoas* on the innominate, prominent third trochanter) with limited lateral rotation capacity (feebly convex ischial spine, lesser trochanter of the femur ventral rather than medial). The femur is abducted, as indicated by the angled pelvic floor of the innominate, the off-centre fovea capitis and the prominent third trochanter. The knee joint is stable (asymmetrical femoral condyles, well-marked femoral lateral insertion of the *M. popliteus*, partially involved in the medial rotation of the tibia, medially twisted femur) and flexed (femoral mediodistal insertion of the medial head of the *M. gastrocnemius* and lateral insertion of the *M. popliteus*).

#### Postcranium of *Apterodon* indet

The humerus and femur of *Apterodon* indet. differ slightly compared to those of *A. langebadreae* nov. sp. The medial rim of the trochlea being less distal than in *A. langebadreae* nov. sp., the elbow joint was certainly more flexible. On the femur, the greater trochanter extends beyond the level of the head, the lesser trochanter is more ventrally located, and the patellar trochlea is mediolaterally narrower, suggesting a more efficient extension of the hindlimb and reduced lateral rotational mobility of the hip compared to the new Libyan species. The morphology of the radius and talus allow to precise the locomotion mode of *Apterodon*. In fact, the deep scar located distolaterally to the radial bicipital tuberosity might be the insertion area of the *M. flexor profundus digitorum*, responsible for the flexion of the wrist and digits. This could be in agreement with the strong medial ridge for this muscle on the ulna of *A. langeabdreae* nov. sp. and *A. macrognathus*. *M. flexor profundus digitorum* probably acted with the powerful *M. abductor pollicis longus* (for which a deep lateral groove is visible on the ulna of *Apterodon* species) to enhance the carpal and digital mobility. The long calcaneal tuber and the short and robust calcaneus distally to the subtalar joint (involving the ectal and sustentacular facets), recall the morphology of fossorial and aquatic mammals. The astragalar trochlea is weakly grooved, which traduces a plantigrade posture, as notably found in scansorial and generalized terrestrial taxa [81]. The dorsal position of the astragalar foramen and the lateral margin of the trochlea extending slightly beyond it suggest limited plantarflexion between the tibia and astragalus at the crurotalar joint ( = upper ankle joint), unlike digitigrade mammals such as modern canids [64]. Several morphological features reduce the range of pedal inversion and eversion compared to scansorial and arboreal carnivorans: the relatively reduced peroneal tubercle on the calcaneus [68], the weakly convex sustentacular facet and slightly spiraled ectal facet at the subtalar joint ( = lower ankle joint), and the weak rotation of the astragalar head relative to the long axis at the transverse tarsal joint. Finally, the likely flattened tail of *Apterodon* indet. could suggest the use of dorsoventral caudal undulation for propulsion during swimming.

#### Paleobiological conclusions

*Apterodon langebadreae* nov. sp. is suggested to be a semi-aquatic hyaenodontidan with substantial ability to dig. These two behaviours are not mutually exclusive and occur together in various degrees, notably in extant otters which build burrows. Forelimb features of the new Libyan species indicate a stable humeroulnar and radioulnar joints, a limited pronation-supination capacity compared to scansorial and arboreal taxa, and a powerful carpal and digital flexion. Hindlimb features suggest a pronounced hip flexion, a strong abduction of the femur, and a stable and flexed knee. Moreover, the laterally curved and dosroventrally thick humerus, the robust and dorsoventral compression of the femur, as well as the possible presence of a flattened tail all unit to suggest an adaptation to swim, maybe invloving hindlimb paddling and dorsoventral caudal undulation. The wide range of abduction at the hip joint could probably allow *Apterodon langebadreae* nov. sp. to walk on uneven substrate when on land [86]. Moreover, the bunodont dental morphology and the very long mandibular ramus which might provide more strength for mastication could indicate that *Apterodon langebadreae* nov. sp. feed on hard items. In fact, *A. langebadreae* nov. sp. occurs in the mixed tidal and storm dominated environement of the Idam Unit of Dur At-Talah (e.g., [36], [42]). The sequence revealed rich bioturbations associated to the ichnogenus *Thalassinoides*, interpreted as burrows of crustaceans (crabs, mud shrimps or prawns) [42], which could constitute a possible diet for the new Libyan species. Thus, *A. langebadreae* nov. sp. seems to be well-adapted for paddling and digging probably to feed on invertebrates along the marine coast of Dur At-Talah. The ecology of *Apterodon* species is probably similar as judged by the morphological resemblances of the cranial and postcranial remains between the Libyan and Egyptian species.

A semi-aquatic locomotor style is unusual in hyaenodontidans (e.g., [87]–[89] and references therein). Semi-fossorial adaptations are only suggested for the North American middle Eocene *Thinocyon* genus and *Limnocyon verus*, and for the European middle Eocene genus *Matthodon* [87], [90]. According to Matthew [91] and Gebo & Rose [81], the postcranial skeleton of *Thinocyon* bears also important similarities both in size and morphology with the semi-aquatic mink *Mustela vison*. However, the morphological features which could likely be linked to digging or swimming behaviour are far less developed in comparison to *Apterodon* (e.g., weak deltopectoral crest on the humerus, long humerus, shorter calcaneal tuber). It is worth noting that, in Africa, the only hyaenodontidan for which postcranial remains have been described so far (apart those of *Apterodon macrognathus*), is *Pterodon africanus* (from the Upper Eocene Fayum Jebel Qatrani Formation) which is supposed to be cursorial [12], [16].

### Phylogenetical analysis

Numerous hyaenodontidan phylogenetic relationships are still debated. Although substantial advances have been recently brought to investigate this issue [4], [18], [23], [52], [56], [90], [92]–[96], the Apterodontinae have never been integrated in a character matrix. Tilden & Holroyd [29] proposed *Apterodon* as close relative or member of hyaenodontines *s.l.* (Hyaenodontinae Leidy, 1869 [58] and Hyainailourinae Pilgrim, 1932 [24] = Pterodontinae Polly, 1996 [23]). Peigné *et al.* [93] and Lewis & Morlo [25] suggested a close relationship with hyainailourines, deriving from an *Arfia*-like ancestor. As Polly [23] demonstrated that the reduction of the metaconid and talonid on lower molars occurred independently in hyaenodontines *s.s.* and hyainailurines, we choose to preliminary investigate the relationships of *Apterodon* within hyainailurines, judging the hyaenodontines too derived to be close relatives. Moreover, Tilden *et al.* [29] and Lange-Badré & Böhme [30] suggested that *Apterodon* originated in Africa, because the European species (*A. gaudryi* and *A. intermedius*) are more recent, less numerous, and seem morphologically more derived than the African ones. Thus, the new discoveries from Dur At-Talah involve a re-examination of the relationships of *Apterodon* species and a preliminary study of the Apterodontinae subfamily relationships within hyaenodontidans.

#### Material and methods

We performed a cladistic analysis based on 17 hyaenodontidan species and 3 outgroup taxa. The ingroup taxa include the Eocene and Oligocene Apterodontinae *Quasiapterodon minutus* (Schlosser, 1911) [16], *Apterodon gaudryi*, *A. macrognathus*, *A. altidens*, *A. saghensis*, *A. langebadreae* nov. sp. from Africa and Europe, the Oligocene and Miocene Hyainailourinae *Metasinopa fraasi* Osborn, 1909 [14], *M. napaki* Savage, 1965 [97], *Dissopsalis carnifex* Pilgrim, 1910 [98], *D. pyroclasticus* Savage, 1965 [97], *Anasinopa leakeyi* Savage, 1965 [97] from Africa and Asia, the Eocene “Proviverrinae” *Kyawadawia lupina* Egi *et al.*, 2005 [96], *Paratritemnodon indicus* Rango Rao, 1973 [99] and *Arfia langebadreae* Lavrov & Lopatin, 2004 [100] from Asia, *Arfia opistothoma* (Matthew, 1901) [101] from North America, *Arfia gingerichi* Smith & Smith, 2001 [102] and *Proviverra typica* Rütimeyer, 1862 [103] from Europe. The specimen of *A. intermedius* is not included in this analysis because of its poor preservation state and questionable dental attribution (as discussed in the systematic part). Operational taxonomic units (OTUs) are scored at the specific level primary to provide information about the phylogenetic relationships of *Apterodon* species and to test the primitive state of African *Apterodon* relative to European ones. The only exception is the outgroup taxon *Prokennalestes*, for which we include two species (*P. trofimovi* and *P. minor* Kielan-Jaworowska & Dashzeveg, 1989 [104]) in order to construct a statifying set of characters for the matrix coding. We choose to use 3 outgroup taxa: the Cretaceous Cimolestidae (sensu Kielan-Jaworowska *et al.* 2004 [105]) *Cimolestes magnus* Clemens & Russell, 1965 [106], which represents the most probable hyaenodontidan ancestral morphotype [107], and *Maelestes gobiensis* Wible *et al.*, 2007 [108], in addition to the Cretacous basal eutherian *Prokennalestes*. *Cimolestes magnus* is a traditionnal outgroup component of the hyaenodontidan phylogenetic studies [18], [23], [56], [92], [94], [96]. Moreover, *Maelestes* and *Prokennalestes* are part of the outgroup taxa chosen for the cladistic analysis of Solé [56].

**Figure 6:**
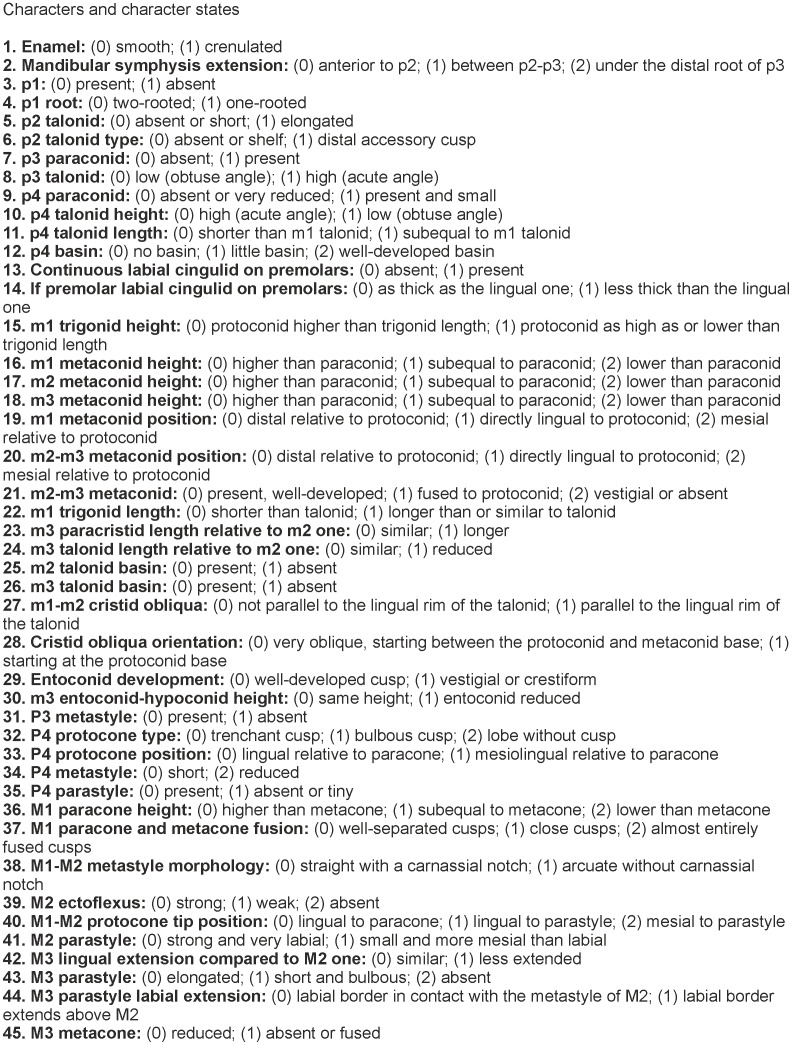
Characters and character states.

We used 45 characters (Figure 6), only referred to dental material because cranial and postcranial data are too poorly known for the analyzed taxa and introduce numerous missing data in the matrix. Of the 45 characters, 11 characters were ordered (2, 12, 16–21, 36, 37, 40). All the characters were unweigthed. The 3 parcimony-uninformative characters are kept to facilitate future analyses incorporating additional taxa into the matrix. We analyzed the matrix using the branch and bound algorithm of PAUP (Phylogenetic Analysis Using Parcimony) version 4.0b10 [109]. Bremer indexes are calculated for each node of the strict consensus and 50% majority rule consensus trees produced. Bootstrap values are reported for the majority consensus tree, when it is not equal at a node to 100%. The matrix is presented in Figure S1.

#### Results and discussion

**Figure 7:**
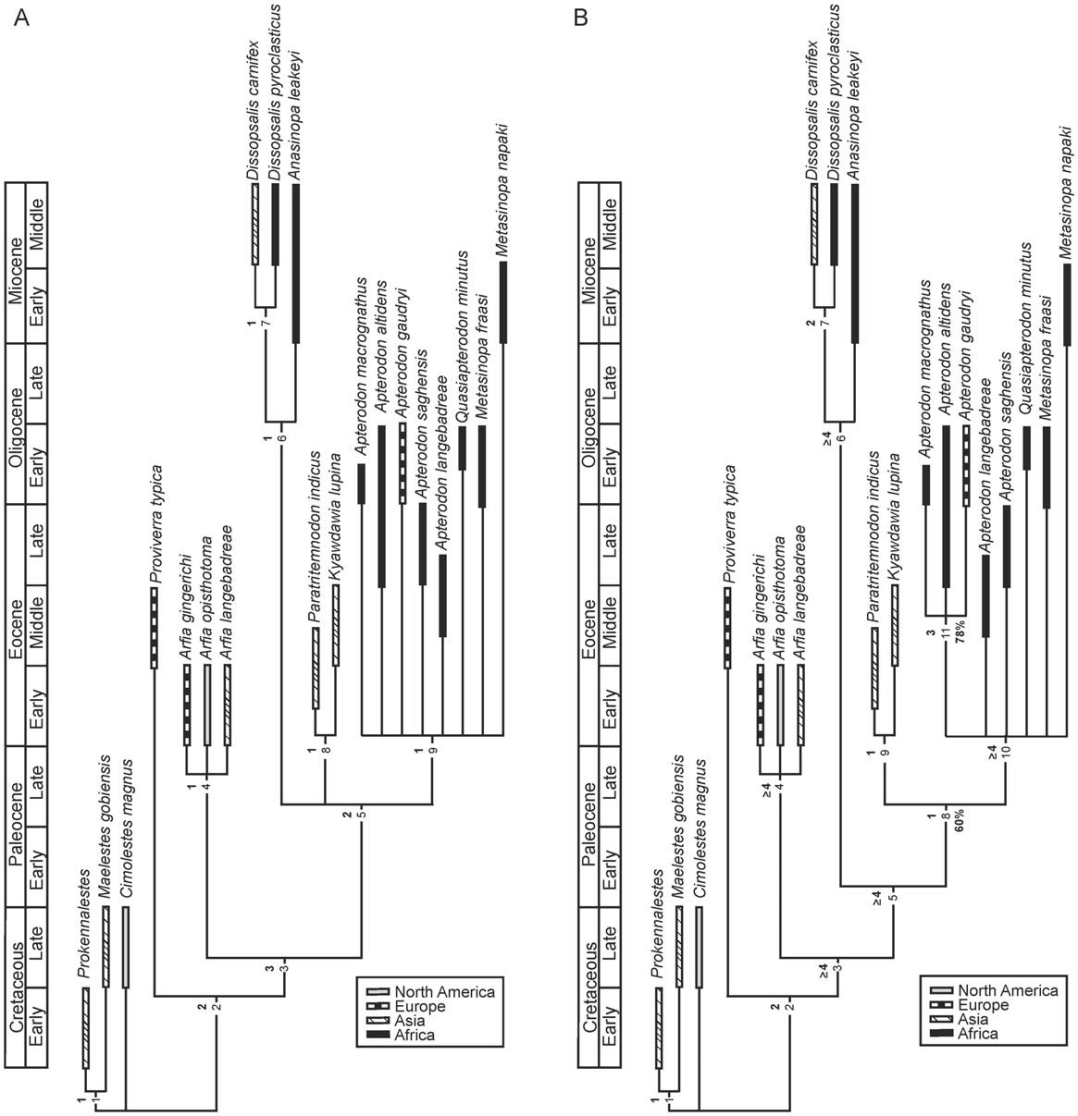
Consensus of the most parsimonious trees obtained by cladistic analysis using branch and bound algorithm of PAUP v4.0b10. Strict consensus tree (103 steps; CI = 0.57; RI = 0.74) in A and 50% majority rule consensus tree (99 steps; CI = 0.6; RI = 0.76) in B. Bremer indexes are noted in bold above the node number for both trees, bootstrap values (when not equal to 100%) are indicated in bold below the node number for the majority consensus.

The parcimony analysis produced 2460 equally parsimonious trees of 95 steps. The strict consensus (103 steps; CI = 0.57; RI = 0.74) and the 50% majority rule consensus (99 steps; CI = 0.6; RI = 0.76) of these trees are presented in figure 7. Bremer support varies from 1 to ≥4 depending on nodes.

Both the strict and majority consensus trees show *Proviverra typica* as the most basal species of our ingroup (node 2 in figure 7A–B). This is in agreement with previous phylogenetical analyses, where European “proviverrines” *Proviverra*-like are considered as bearing the most primitive morphotype among hyaenodontidans (e.g., [56], [92], [96]). Moreover, *Proviverra typica* appears sister taxon of a relatively well-supported clade comprising *Arfia*, *Anasinopa*, *Dissopsalis*, *Paratritemnodon*, *Kyawdawia*, *Metasinopa*, *Quasipaterodon* and *Apterodon* (node 3 in figure 7A–B). This node is supported by the presence of a crenulated enamel (char. 1(1)), a one-rooted p1 (char. 4(1)), a little basin on p4 (char. 12(1)), a m1 trigonid height subequal or lower to the trigonid length (char. 15(1)), a m3 metaconid lower than the paraconid (char. 18(2)), a m1 trigonid longer than or similar in length to the talonid (char. 22(1)), a m3 paracristid longer than the m2 one (char. 23(1)), a cristid obliqua starting at the base of the protoconid on lower molars (char. 28(1)), a reduced m3 entoconid (char. 30(1)), a bulbous P4 protocone (char. 32(1)), which is mesiolingually placed with respect to the paracone (char. 33(1)), and a weak M2 ectoflexus (char. 39(1)). The characters 15, 18, and 28 have a consistency index of 1.

The genus *Arfia* is monophyletic (node 4 in figure 7A–B) and is characterized by a m1 and m2 metaconid higher than the paraconid (char. 16(0), 17(0)), a m1 metaconid distal relative to the protoconid (char. 19(0)), and an arcuate morphology without carnassial notch of M1–M2 metastyle (char. 38(1); CI = 1). The *Arfia* species are basal to a clade including a set of hyainailourines, Southern Asian “proviverrines” and apterodontines (node 5 in figure 7A–B). Polly [23] proposed for the first time a close relationship between *Arfia* and hyainailourines, with *Arfia* appearing basal to hyainailourines (i.e., *Pterodon*, *Hyainailouros*, *Dissopsalis*). This hypothesis was later followed by Peigné *et al.* [93] and Lewis & Morlo [25]. Moreover, Peigné *et al.* [93] proposed an *Arfia*-like ancestor for both hyainailourines and Southern Asian “proviverrines” (*Paratritemnodon*, *Kyawdawia* but also *Yarshea*). After Lewis & Morlo [25], these Southern Asian “proviverrines” could constitute an endemic lineage evolving independently from African and European “proviverrines” during the Paleogene, whereas for Holroyd [18] and Egi *et al.* [96], they are part of an Afro-asian clade including the hyainailourines *Dissopsalis*, *Metasinopa* and *Anasinopa*. In our study, the hyainailourines appear polyphyletic and the Southern Asian “proviverrines” and afro-european apterodontines could be comprised in this subfamily deriving from an *Arfia*-like ancestor. The relationship underlined by the node 5 is supported by a p4 paraconid absent or very reduced (char. 9(0)), a m1 and m2 metaconid lower than the paraconid (char. 16(2), 17(2)), M1–M2 protocone lingually placed with respect to the parastyle (char. 40(1)), and a M3 metacone absent or fused to the paracone (char. 45(1)). Non homoplasious characters are the presence of a p2 talonid shelf (char. 6(0)), a continuous labial cingulid on premolars (char.13(1)), m1–m2 cristid obliqua parallel to the lingual rim of the talonid (char. 27(1)), a crestiform or vestigial entoconid on lower molars (char. 29(1)), and close M1 paracone and metacone (char. 37(1)). The node 5 of the strict consensus tree is also supported by a tiny or absent P4 parastyle (char. 35(1)).

*Dissopsalis* and *Anasinopa* (node 6 in figure 7A–B) share a two-rooted p1 (char. 4(0)), a well-developed p4 basin (char. 12(2)), a M1 paracone lower than the metacone (char. 36(2)), a M1–M2 protocone mesially displaced relative to the parastyle (char. 40(2)), and a short and bulbous M3 parastyle (char. 43(1)), the character 36 having a consistency index of 1. On the strict consensus, these two genera also share the presence of a P4 parastyle (char. 35(0)). Savage [97] described the holotype and only specimen of *Dissopsalis pyroclasticus* as bearing a single-rooted p1, even if the alveolar region between p1 and canine was not well-preserved. The examination of a mandible of *D. carnifex* (GSP-Y51401), which presents two alveoli for p1 and the same premolar alveoli configuration as the *D. pyroclasticus* holotype, could indicate that both species are characterized by a two-rooted p1.

*Anasinopa leakeyi* appears more primitive than *Dissopsalis*, as proposed by Barry [92] and Egi *et al.* [96]. *Dissopsalis* species (node 7 in figure 7A–B) display a p3 paraconid (char. 7(1)), m2–m3 metaconids fused to the protoconid (char. 21(1)), M1 paracone and metacone almost entirely fused (char. 37(2)) and lack a M2 ectoflexus (char. 39(2)). Only the character 37 is not homoplasious. The reduction of metaconids and the close paracone and metacone on upper molars in *Dissopsalis* suggest a more hypercarnivorous diet compared to *Anasinopa*.

The close relationship between the two Southern Asian “proviverrine” species *Paratritemnodon indicus* and *Kyawdawia lupina* (node 8 in figure 7A and node 9 in figure 7B) is defined by the presence of a p3 paraconid (char. 7(1)), a labial cingulid as thick as the lingual one on premolars (char. 14(0)), a trenchant P4 protocone (char. 32(0)), a strong M2 ectoflexus (char. 39(0)) and a M3 parastyle extending more labially than M2 (char. 44(1)), this last character having a consistency index of 1. In Egi *et al.* [96], *Paratritemnodon* and *Kyawdawia* also belong to the same clade, but together with the Afro-arabian Paleogene hyainailourine *Masrasector*. According to our results, the Southern Asian taxa could have evolved from an *Arfia*-like ancestor, independently (figure 7A) or not (figure 7B) from the Afro-european hyainailourine and apterodontine radiation. The relationship of these Asian species with African hyainailourines (*Dissopsalis*, *Anasinopa*, *Metasinopa*) and apterodontines (*Apterodon*, *Quasiapterodon*) is however unclear. The node 8 in the majority consensus tree is poorly supported and only defined by homoplasious characters: a mandibular symphysis extending under the distal root of p3 (char. 2(2)), a low p3 talonid (char. 8(0)), a P4 protocone lobe (char. 32(2)) and a tiny P4 parastyle (char. 35(1)).

Both the strict and majority consensus trees show a polyphyly of Apterodontinae relative to the position of *Metasinopa* species (node 9 in figure 7A and node 10 in figure 7B). This clade regrouping *Apterodon*, *Quasiapterodon* and *Metasinopa* is supported by a low p3 talonid (char. 8(0)), the lack of p4 talonid basin (char. 12(0)), a m1 metaconid mesially placed with respect to the protoconid (char. 19(2); only known in *Apterodon macrognathus*), a vestigial or absent m2–m3 metaconid (char. 21(2)), the lack of m3 talonid basin (char. 26(1)), the lack of P3 metastyle (char. 31(1)), a P4 protocone lobe without cusp (char. 32(2)), a M1–M2 protocone directly lingual to the paracone (char. 40(0)). The strict consensus also indicates a mandibular symphysis extending under the distal root of p3 (char. 2(2)) as a synapomorphy of the clade. Except the character 31, the others are homoplasious. Even if *Apterodon* and *Quasipaterodon* do not appear monophyletic in our analysis, it is clear that they are morphologically different from the *Metasinopa* species, particularly in the more extreme reduction of the metaconid on lower molars (derived character) and the presence of a P4 protocone lobe without cusp for *Apterodon* species. Therefore, *Metasinopa* could be the closest relative genus to apterodontines.

Within apterodontines, *Quasiapterodon minutus*, *Apterodon saghensis* and *A. langebadreae* nov. sp. are basal to a clade including *A. macrognathus*, *A. altidens* and *A. gaudryi* in the majority consensus tree (node 11 in figure 7B). This relationship is supported by a low p4 paraconid (char. 10(1)), the lack of m2 talonid basin (char. 25(1); CI = 1), a reduced P4 metastyle (char. 34(1)) and a short M3 metastyle (char. 43(1)). *A. langebadreae* nov. sp. may be the most primitive *Apterodon* species, the basal position of *A. saghensis* (the oldest *Apterodon* species of the Fayum) being weakened by the poor fossil record available for the matrix coding (notably the lack of lower molars and upper dental specimens).

Our analysis reveals complex paleobiogeographical scenarii from the Eocene to the Miocene between Europe-North America-Asia, Africa-Asia and Africa-Europe. While the oldest hyainailourines could be European (*Francotherium lindgreni* from the early Eocene of Western Europe [25]) or African (?early Eocene from Gour Lazib [10]), our analysis suggests that this subfamily could derive from an *Arfia*-like ancestor, which geographical distribution is holarctic during the early Eocene, and that hyainailourines could include the Southern Asian “proviverrines” *Paratritemnodon* and *Kyawdawia*, as well as apterodontines. These relationships implicate notably dispersal events between Southern Asia and Africa possibly during the early Eocene, an hypothesis previously formulated by Holroyd [18] and Egi *et al.* [96]. Concerning *Apterodon*, Simons & Gingerich [61] first suggested that this genus could have evolved in isolation in Africa and dispersed around the Tethys Sea to Europe in the early Oligocene. They based this hypothesis on the oldest occurrence of the group (in Africa) and on the richest specimens from the Fayum compared to those from Europe. Lange-Badré & Böhme [30] also proposed an Asian origin for *Apterodon* followed by an Eocene immigration event into Africa and then a dispersion episode in the early Oligocene or nearly before towards Europe. According to our analysis, *Apterodon* and *Quasiapterodon* seem to originate in Africa, from an ancestor morphologically close to the Oligo-Miocene genus *Metasinopa*. The derived position of *A. gaudryi* within *Apterodon* species could support the hypothesis primary expressed by Simons & Gingerich [61] of an African origin of this genus and a migration towards Europe between the Late Eocene and early Oligocene to give rise to the European species [3], [30].

## Supporting Information

Figure S1 **Character state data matrix.** “?”: missing data; “−”: inapplicable.
(TIF)

Table S1 **Measurements (in cm) and indexes of the main postcranial elements of the studied Hyaenodontida.** Index 1 = mediolateral width of the humeral distal epiphysis/humerus length; index 2 = maximum proximodistal length of the olecranon/ulna length (see [73]). Asterisks indicate estimations due to broken and well-worn parts. Measurements are from first-hand.
(TIF)
