# Supplementary figures and images for: New Apterodontinae (Hyaenodontida) from the Eocene Locality of Dur At-Talah (Libya): Systematic, Paleoecological and Phylogenetical Implications
Source: PLoS One. 2012 Nov 21;7(11):e49054. doi: 10.1371/journal.pone.0049054 (PMC3504055; doi:10.1371/journal.pone.0049054)

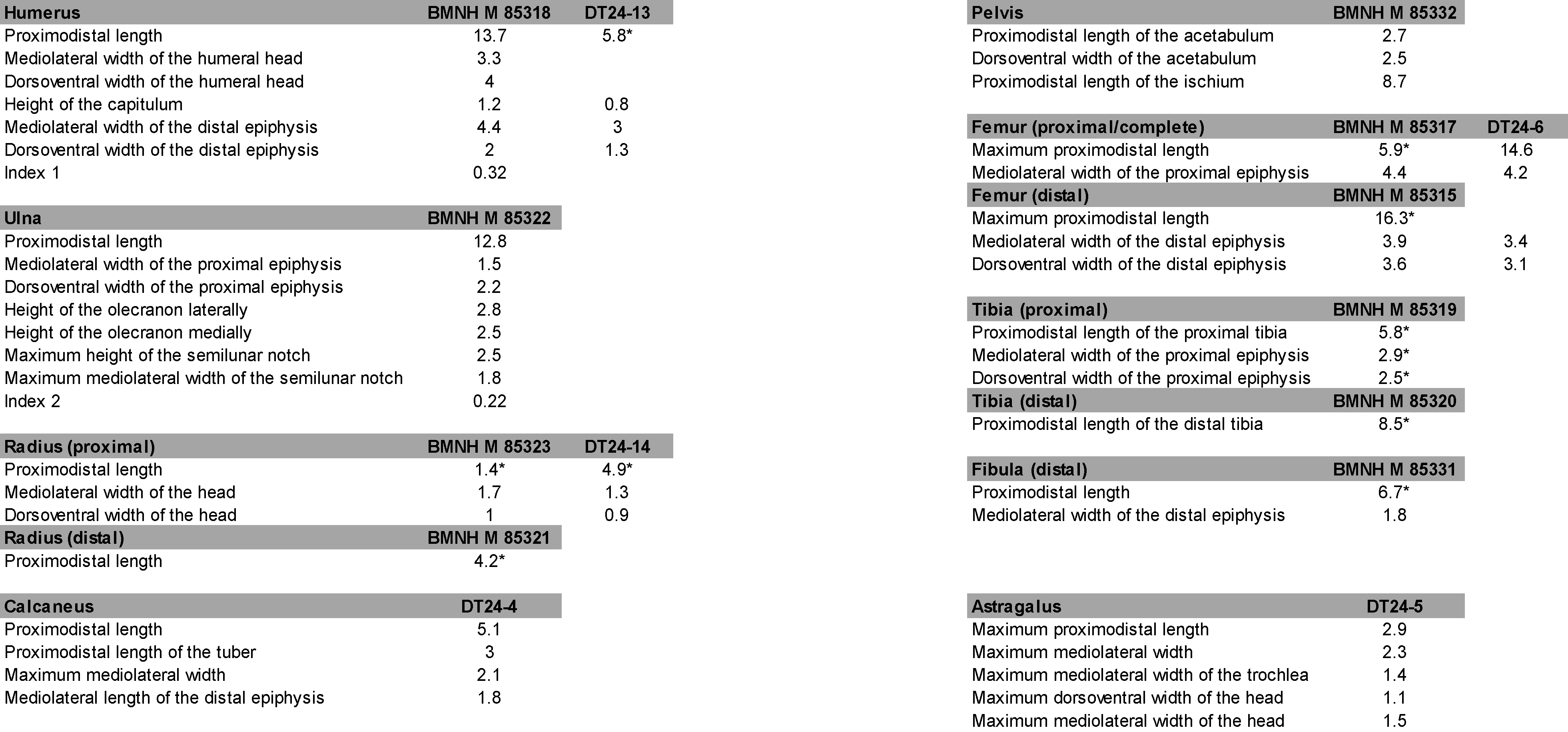

Supplement: Table S1 — Measurements (in cm) and indexes of the main postcranial elements of the studied Hyaenodontida. Index 1 = mediolateral width of the humeral distal epiphysis/humerus length; index 2 = maximum proximodistal length of the olecranon/ulna length (see [73]). Asterisks indicate estimations due to broken and well-worn parts. Measurements are from first-hand. (TIF) [file pone.0049054.s002.tif]
